# Publisher Correction: Reconstruction of proto-vertebrate, protocyclostome and proto-gnathostome genomes provides new insights into early vertebrate evolution

**DOI:** 10.1038/s41467-021-25110-8

**Published:** 2021-07-29

**Authors:** Yoichiro Nakatani, Prashant Shingate, Vydianathan Ravi, Nisha E. Pillai, Aravind Prasad, Aoife McLysaght, Byrappa Venkatesh

**Affiliations:** 1 Smurfit Institute of Genetics, Trinity College Dublin, University of Dublin, Dublin, 2 Ireland; 2 Comparative and Medical Genomics Laboratory, Institute of Molecular and Cell Biology, A*STAR, Biopolis, Singapore, Singapore; 3 Department of Paediatrics, Yong Loo Lin School of Medicine, National University of Singapre, Singapore, Singapore; 4 Present Address: Graduate School of Medicine, Osaka University, Osaka, Japan

**Keywords:** Molecular evolution, Genome evolution, Polyploidy

Correction to: *Nature Communications* 10.1038/s41467-021-24573-z, published online 23 July 2021.

The original version of this Article contained an additional reference and incorrectly reported the number of genes in the penultimate paragraph of the ‘Introduction’. The original sentence read “… compared to 12,137 human genes in refs. ^8, 13^, 434 human genes in ref. ^15^”. This has been corrected to “… compared to 12,137 human genes in ref. ^13^, and 8,434 human genes in ref. ^15^”. This has been corrected in the PDF and HTML versions of the Article.

