# Supplementary material for: Reconstruction of proto-vertebrate, proto-cyclostome and proto-gnathostome genomes provides new insights into early vertebrate evolution
Source: Nat Commun. 2021 Jul 23;12:4489. doi: 10.1038/s41467-021-24573-z (PMC8302630; doi:10.1038/s41467-021-24573-z)
Supplement: Supplementary file 1 — Supplementary Information [file 41467_2021_24573_MOESM1_ESM.pdf]

Supplementary Information

for

Reconstruction of proto-vertebrate, proto-cyclostome and proto-gnathostome genomes provides new insights into early vertebrate evolution

Nakatani et al.

Contents

Supplementary Note 1. Sequencing, assembly and annotation of elephant shark and Japanese lamprey genomes 4
1.1 Elephant shark genome assembly 4
Single-molecule, real-time (SMRT) sequencing 4
Whole-genome shotgun sequencing using Illumina 4
Contig-level whole-genome assembly 4
Hi-C aided chromosome-level whole-genome assembly 5
Assembly quality and completeness 6
Repetitive sequences 7
Genome annotation 7
1.2 Japanese lamprey genome assembly 11
Single-molecule, real-time (SMRT) sequencing 11
Whole-genome shotgun sequencing using Illumina 11
Estimation of genome size and heterozygosity 11
Contig-level whole-genome assembly 12
Hi-C aided chromosome-level whole-genome assembly 13
Assembly quality and completeness 14
Repetitive sequences 15
Genome annotation 15
Supplementary Note 2. Annotation of orthologues and paralogues 19
2.1 Orthologues between vertebrates and invertebrates 19
2.2 Orthologues between cyclostomes and gnathostomes 19
2.3 Orthologues between sea lamprey and Japanese lamprey 19
2.4 Cyclostome paralogues 20
2.5 Elephant shark paralogues 20
Supplementary Note 3. Reconstruction analysis 20
3.1 Reconstruction of the proto-vertebrate genome 20
3.2 Reconstruction of the proto- 23
gnathostome genome 23
3.3 The proto-cyclostome genome was shaped by six-fold genome duplication. 24
Supplementary Note 4. Evolution of proto-gnathostome and proto-cyclostome chromosomes 24
4.1 Previous arguments on the origin of microchromosomes in gnathostomes 24
4.2 Potential factors affecting the rearrangement rate of microchromosomes 25

4.3 Mechanisms of chromosome fusions between 1R and 2R 26
4.4 Inferred evolutionary scenario and biased gene retention 26
4.5 Functional biases between the two proto-gnathostome subgenomes 27
Supplementary Note 5. Gene tree analysis 29
5.1 Classification of duplication timing 29
Supplementary Figures 33
Supplementary References 49

Supplementary Note 1. Sequencing, assembly and annotation of elephant shark and Japanese lamprey genomes

1.1 Elephant shark genome assembly

Single-molecule, real-time (SMRT) sequencing

For SMRT sequencing, high-molecular-weight genomic DNA was extracted from the testis of a single elephant shark collected in Hobart, Tasmania, Australia. This DNA was used to prepare long insert genomic libraries followed by SMRTbell^{TM} templates. The PacBio RS-II platform was used to sequence the SMRTbell^{TM} templates using multiple sequencing chemistries generating ~69 Gb of sequence data (~69× genome coverage of the estimated 1 Gb genome[1]) (Supplementary Table 1).

Supplementary Table 1. SMRT sequencing statistics for the elephant shark genome.

Chemistry	Number of reads	N50 read length (bp)	Total read length (Gb)	Fold-coverage
P4-C2	781,145	5,392	3.26	3.26
P5-C3	1,460,752	8,588	8.88	8.88
P6-C4	6,275,480	13,315	56.7	56.7
Total	8,517,377		68.84	68.84

Whole-genome shotgun sequencing using Illumina

For whole-genome shotgun sequencing, PCR-free libraries with insert sizes ranging from 350 to 490 bp were prepared using TruSeq DNA PCR-free kit (Illumina, San Diego, CA, USA) and Kappa Hyper Prep kit (Kapa Biosystems, South Africa). These libraries were sequenced on the Illumina HiSeq 2500 and 4000 platforms to generate ~100 Gb of 150 bp paired-end reads amounting to ~100× coverage of the genome.

Contig-level whole-genome assembly

The SMRT reads were assembled using the FALCON Assembler v0.3.0[2] with the help of DNAnexus, Inc. (San Francisco, CA, USA). The assembled contigs were polished using raw SMRT reads with Quiver[3]. This was followed by additional polishing of the assembly using ~100 Gb of Illumina paired-end shotgun reads with Pilon v1.20[4]. This process generated a

Primary assembly spanning 1.05 Gb and an Accessory (heterozygous) assembly spanning 253 Mb. Only the Primary assembly was used for extending the contiguity of the assembly. The Primary assembly is supposed to contain only unique contigs. However, we found several instances of duplicate (heterozygous) contigs in this assembly. In addition, several contig pairs were found to overlap by 10 kb to ~2 Mb at the terminal region. An in-house script was used to identify and remove duplicate contigs with criteria of ≥95% identity and coverage of ≥80% with respect to the shorter contig. Another in-house script was used to merge contigs that showed at least 30 kb overlap at the ends with ≥95% identity.

Hi-C aided chromosome-level whole-genome assembly

A Dovetail Chicago® library (Dovetail Genomics, Santa Cruz, CA) was prepared using high molecular weight DNA sample and a proximity ligation technology. A total of 152 million read pairs of length 100 bp were generated from this library. The FALCON non-redundant Primary assembly was scaffolded using Dovetail Chicago library reads with the HiRise scaffolding program[5] resulting in an intermediary assembly. For further extending the contiguity, a Dovetail Hi-C library was generated using the Dovetail™ Hi-C kit and frozen testis tissue from the elephant shark. The second round of scaffolding was performed using 521 million Dovetail Hi-C library read pairs of length 151 bp. The scaffolds built using Hi-C library were subjected to one round of gap-filling using all error-corrected SMRT reads and PBJelly program[6] resulting in the final PacBio-HiC assembly. The statistics of the previously published 454-assembly[1] and the current "PacBio-HiC" assembly are given in Supplementary Table 2.

Supplementary Table 2. Assembly statistics of the published 454- assembly[1]and the current PacBio-HiC assembly of the elephant shark genome.

	**Published 454-assembly[1]**	**PacBio-HiC assembly**
Assembled genome size	974.4 Mb	991.5 Mb
Number of contigs	67,425	3,865
Contig N50 length	46.6 kb	1.6 Mb
Longest contig length	631 kb	9.5 Mb
Number of scaffolds	21,208	1,761
Scaffold N50 length	4.5 Mb	69.3 Mb
Longest scaffold length	18.5 Mb	139.2 Mb
Total gap length in the assembly	37.5 Mb	69.3 kb

Assembly quality and completeness

In order to assess the quality of the PacBio-HiC assembly, 76,126 BAC-end read pairs, generated previously from an elephant shark BAC library (average insert size of ~150 kb) using DNA from the same male individual[1], were aligned to the assembly using BLASTN from BLAST+ v2.5.0 package. 54,546 BAC-end read pairs aligned uniquely to the assembly with 5,233 pairs mapping to a single scaffold while the remaining 1,538 pairs mapped to different scaffolds. The latter (147 scaffolds in total) represent potential Hi-C mis-assemblies. However, 56 of these scaffolds are shorter than 100 kb indicating that there are no large-scale mis-assembled scaffolds in the assembly. Note that our reconstruction analysis is unlikely to be affected by such assembly errors (false joining), because we made synteny blocks by comparing elephant shark scaffolds with several gnathostome genomes. As a further evaluation of the assembly quality, a previously sequenced set of 76 complete BAC clones[1] were aligned to the assembly using BLASTN. Out of 76 BACs, 74 (97%) aligned completely to a single scaffold each while the remaining two aligned to more than one scaffold. These data show that there are no large-scale structural mis-assemblies in the PacBio-HiC assembly. The overall contiguity of the present assembly is better than that of the previously published 454-assembly[1] of the elephant shark (Supplementary Fig. 1a). The completeness of the assembly was assessed by searching for 2,586 vertebrate genes set from OrthoDB v9 of the Benchmarking Universal Single-Copy Orthologs (BUSCO v2.0[7]) in the assembly. The PacBio-HiC elephant shark genome assembly contained complete sequences for 90.3% of these genes and partial sequences for 4.3% of genes, while the remaining genes were missing from the assembly. Assembly completeness was further evaluated using TRINITY-based transcriptomes that were generated previously from 10 tissues[1]. The TRINITY transcripts were subjected to CD-HIT v4.6.1[8] clustering using 97% identity and 80% coverage cut-offs, and resultant transcripts that were ≥1000 bp (~67,000) were aligned to the assembly using BLAT v35[9]. The number of transcripts aligned was computed using an in-house Perl program. Approximately 91% of the transcripts were found to align to the assembly (≥90% coverage and ≥90% sequence identity) indicating that the assembly contains most of the gene sequences.

Repetitive sequences

RepeatModeler v1.0.10[10] was used to generate a *de novo* repeat library from the elephant shark PacBio-HiC assembly. Repetitive elements of the unknown class were classified using the TEclass v2.1.3 program[11]. This repeat library was combined with known elephant shark repeats from RepBase v22.05[12]. The combined repeat library was then clustered using CD-HIT[8] with 94% as identity cut-off and 80% as coverage cut-off. The longest repeat sequence from each cluster was chosen to generate a non-redundant repeat library. These repeat sequences were screened against human proteins obtained from RefSeq release 84[13, 14] using BLASTX[15, 16] with an E-value cutoff of 10^{-20} and repeat sequences showing significant similarity were removed from the library. The final elephant shark genome-specific repeat library contained 1,869 repetitive elements. These elements were used to mask repeats in the PacBio-HiC assembly.

Genome annotation

Genome annotation was performed using the MAKER pipeline v2.31.8[17]. The repeat library (see previous section) was used as input to mask repetitive regions in the genome assembly using RepeatMasker v4.0[18]. Supplementary Table 3 summarizes different types of repetitive sequences in the elephant shark genome assembly. Approximately ~42% of the elephant shark genome consists of repetitive sequences which is substantially higher than that predicted in the previous 454-assembly[1] (28%) and reflects the higher level of contiguity of the PacBio-HiC assembly.

We performed both evidence-based gene prediction and AUGUSTUS v 3.2.1[19]-based *ab initio* gene prediction. For evidence-based annotation, we used a CD-HIT[8] clustered set of ~67,000 transcript sequences with length ≥1 kb from a transcriptome assembly generated from 10 different tissues of elephant shark and two tissues from nurse shark (*Ginglymostoma cirratum*) in a previous study[1]. In addition, a combined dataset of ~197,000 RefSeq[13] proteins from *Strongylocentrotus purpuratus*, *Homo sapiens*, *Ciona intestinalis*, *Branchiostoma floridae*, *Danio rerio*, *Oryzias latipes*, *Xenopus tropicalis*, *Gallus gallus*, *Nematostella vectensis* and Elasmobranchii, and 17,772 protein sequences predicted in the 454-assembly[1] were also used.

For the AUGUSTUS[19]-based *ab initio* gene prediction, the transcript and protein sequence-based hint files in the GFF3 format were used as input to aid the gene prediction process as well as to calculate the Annotation Edit Distance (AED) score[17]. The AED score measures the fitness of the predicted gene models to the available evidence such as known transcripts and/or protein sequences supporting it. In the AUGUSTUS[19]-based method, 19,330 genes were predicted. The gene models from both the approaches were merged using an in-house Perl script and a final set of 18,747 protein-coding genes with an AED score ≤0.5 were predicted.

Supplementary Table 3. Repetitive sequences in the elephant shark genome assembly.

Order	Subclass	Count	bpMasked	%masked
DNA		96,791	13,423,679	1.27%
	Academ	33	22,275	0.00%
	Academ-1	320	44,562	0.00%
	CMC-Chapaev-3	1,561	647,329	0.06%
	CMC-EnSpm	5,334	955,840	0.09%
	Crypton-V	65	18,604	0.00%
	Ginger	12,144	4,466,495	0.42%
	IS3EU	9	1,571	0.00%
	Kolobok-Hydra	14	9,618	0.00%
	MULE-MuDR	11	1,885	0.00%
	Maverick	46	9,599	0.00%
	Novosib	312	126,094	0.01%
	PIF-Harbinger	11	3,255	0.00%
	PIF-Spy	106	27,499	0.00%
	PiggyBac	6	801	0.00%
	TcMar-Fot1	9	409	0.00%
	TcMar-Mariner	138	40,237	0.00%
	TcMar-Pogo	4,966	781,152	0.07%
	TcMar-Tc1	4,484	1,531,586	0.14%
	TcMar-Tc2	438	53,618	0.01%
	TcMar-Tigger	4,825	1,145,770	0.11%
	Zator	1,636	463,618	0.04%
	Zisupton	1,488	331,977	0.03%
	hAT	369	177,762	0.02%
	hAT-Ac	2,172	776,881	0.07%
	hAT-Blackjack	833	169,015	0.02%

	hAT-Charlie	5,234	1,141,368	0.11%
	hAT-Tip100	2,255	517,720	0.05%
	hAT-hATx	147	36,369	0.00%
LINE		116,653	17,016,587	1.61%
	CR1	343,817	61,980,704	5.87%
	CR1-Zenon	2,063	449,052	0.04%
	Dong-R4	1,052	185,178	0.02%
	I-Jockey	1,648	271,491	0.03%
	Jockey	1,753	388,774	0.04%
	L1	350	119,524	0.01%
	L1-Tx1	1,041	569,007	0.05%
	L2	531,309	107,849,103	10.21%
	Penelope	54,034	9,976,912	0.94%
	R1	2	377	0.00%
	RTE-BovB	7,473	2,828,905	0.27%
	RTE-RTE	16,513	2,626,344	0.25%
	RTE-X	2,469	642,316	0.06%
LTR		72,264	15,028,787	1.42%
	Copia	120	20,630	0.00%
	ERV1	2,578	1,388,164	0.13%
	ERVK	20	1,130	0.00%
	Gypsy	12,819	7,288,340	0.69%
	Gypsy-Cigr	894	1,312,088	0.12%
	Ngaro	14,196	2,752,816	0.26%
	Pao	333	263,106	0.02%
RC	Helitron	9,049	2,474,772	0.23%
Retro		87955	17,136,750	0
SINE		14001	2,659,380	0
	5S-Deu-L2	5,090	404,106	0.04%
	5S-RTE	826	57,498	0.01%
	ID	12,571	802,468	0.08%
	MIR	404	30,599	0.00%
	U	200	35,009	0.00%
	tRNA	20,873	3,721,109	0.35%
	tRNA-Core	5,938	1,056,228	0.10%

	tRNA-Deu	5,915	799,105	0.08%
	tRNA-Deu-L2	787,843	116,499,218	11.03%
	tRNA-L2	112,690	13,308,243	1.26%
	tRNA-V	3,389	329,360	0.03%
Unknown		23,234	3,678,018	0.35%
Total Interspersed Repeats		2,419,136	422,877,786	40.20%
Low_complexity		39,791	3,439,130	0.33%
	Satellite	10,785	2,589,467	0.25%
	Simple_repeat	278,157	18,610,290	1.76%
	rRNA	958	222,704	0.02%
	snRNA	100	14,235	0.00%
**Total**		2,748,927	447,753,612	42.38%

1.2 Japanese lamprey genome assembly

Single-molecule, real-time (SMRT) sequencing

Japanese lamprey (*Lethenteron japonicum*; also known as the Arctic lamprey *Lethenteron camtschaticum*) were collected from Ishikari River, Hokkaido, Japan. A PacBio-HiC assembly was generated using a protocol similar to the one used for elephant shark. Briefly, high-molecular-weight genomic DNA was extracted from a mature testis (completely filled with sperm) and used for generating SMRT sequences. A total of ~125 Gb of SMRT sequences (~87.8× genome coverage of the estimated 1.43 Gb genome) were generated using the PacBio RS II and Sequel platforms and multiple sequencing chemistries (Supplementary Table 4).

Supplementary Table 4. SMRT sequencing statistics for the Japanese lamprey genome.

Platform	Chemistry	Number of reads	N50 read length (bp)	Total bp in reads (Gb)	Coverage (×)
RS II	P4C2	1,404,069	6,625	7.01	4.90
	P5C3	1,644,648	7,748	9.73	6.80
	P6C4	9,765,783	15,123	89.76	62.77
Sequel	P6C4	2,099,153	14,357	19	13.29
	Total	12,814,500		125.60	87.76

Whole-genome shotgun sequencing using Illumina

The PCR-free libraries were sequenced on an Illumina HiSeq 2000 platform to generate ~93 Gb of 100 bp paired-end reads amounting to ~65× coverage of the genome.

Estimation of genome size and heterozygosity

The Illumina reads with a combined length of 93 Gb were used as input for calculating the distribution of k-mer copy number (KCN). KCN is the frequency of occurrence of all distinct k-mers in the given k-mer dataset. Jellyfish version 2.2.3[20] was employed to calculate KCN distribution at k-mer value k=25 (Supplementary Fig. 2). The genome size was estimated using the Lander-Waterman algorithm (Equation 1 and 2) which explains the relation between estimated read-depth and genome size. In the first step, read-depth (RD) is calculated using P which is the highest occurrence of KCN in the distribution, average read length (r) and k-mer value (k) and then the genome size is estimated as a ratio of the total read length (L) and RD. All

KCN values smaller than the first minima represent random sequencing errors and hence all k-mers below the minima were excluded during genome size estimation. In this case the first minimum is at KCN=11, hence we estimated the percentage error by computing the ratio of area under the curve before the first minima to the total area under the curve. The percentage error was 0.5% and it was subtracted from the estimated genome size.

Thus, using this process, we estimated 1.43 Gb as the genome size of Japanese lamprey (Supplementary Table 5).

$$\mathrm{RD} = P.\frac{r}{(r-k+1)} \quad \ldots\ldots\ldots\ldots\ldots\ldots\ldots\ldots\ldots\ldots\ldots\ldots\ldots\ldots 1)$$

$$\text{Estimated Genome Size } (E) = \frac{\text{Total of Read Lengths } (L)}{\mathrm{RD}} \quad \ldots\ldots\ldots\ldots\ldots\ldots 2)$$

Supplementary Table 5. k-mer values and estimated genome size of the Japanese lamprey

k-mer (k)	Peak (P)	Read Depth (RD)	Estimated Genome size (E) in Gb	First minima	%Error	Corrected Genome size (E) in Gb
25	49	64.27	1.45	11	0.5	1.431

The k-mer plot shows two distinct peaks (bimodal distribution) which indicate a high level of heterogeneity (Supplementary Fig. 2). In a k-mer plot, the first peak and second peak represent k-mer populations from heterozygous and homozygous genomic regions respectively. Hence the percentage heterozygosity of the genome is the ratio of area under first peak to the total area under the entire graph. The k-mer copy number (KCN) values range from 11 to 35 for first peak while KCN values range from 36 to 80 for the second peak. The percentage heterozygosity was calculated using equation 3, where $n1$ and $n2$ are k-mers from the first and second peak, respectively; $k_{n1}$and k_{n2} represent KCN values of $n1$ and $n2$, respectively; and o_{n1} and o_{n2} represent occurrence of $n1$ and $n2$ respectively. The estimated level of heterozygosity is 0.78%.

$$\%\text{heterozygosity} = 100 * \frac{\frac{\sum_{n1=17}^{33}(k_{n1}o_{n1})}{\mathrm{L}}}{\sum_{n1=17}^{33}(k_{n1}o_{n1})+\sum_{n2=34}^{77}(k_{n2}o_{n2})} \quad \ldots\ldots\ldots\ldots\ldots\ldots\ldots\ldots\ldots 3)$$

Contig-level whole-genome assembly

The FALCON Assembler v0.3.0[2] was used to assemble SMRT reads with the help of DNAnexus, Inc. (San Francisco, CA, USA). Resultant contigs were polished using Quiver[3] and

Arrow programs with raw SMRT reads generated from RSII and Sequel platforms, respectively. Further error correction of the assembly was done by using ~93 Gb of Illumina paired-end shotgun reads and Pilon[4]. This procedure resulted in a Primary assembly with total length of 1.25 Gb and an Accessory (heterozygous) assembly spanning 297 Mb. The Primary assembly was used for extending the contiguity of the assembly. Similar to the Primary SMRT assembly of the elephant shark genome, this assembly also contained several duplicate (heterozygous) contigs, and contig pairs with overlapping terminal regions (10 kb to ~2 Mb). Using an in-house script, duplicate contigs that were ≥95% identical with a coverage of ≥80% with respect to the shorter contig were removed. Another in-house script was used to join overlapping contigs that showed at least 30 kb overlap at the ends with ≥95% identity.

Hi-C aided chromosome-level whole-genome assembly

A high molecular weight DNA sample was used to prepare a Dovetail Chicago® library (Dovetail Genomics, Santa Cruz, CA) using the proximity ligation technology. Altogether 133 million read pairs of length 100 bp were sequenced using this library. The non-redundant Primary assembly was scaffolded using Dovetail Chicago library reads and the HiRise scaffolding program[5] resulting in an intermediary assembly. The contiguity was further improved using a Dovetail Hi-C library which was generated using the Dovetail™ Hi-C kit and frozen testis tissue from the Japanese lamprey. This library contained 470 million read pairs of length 151 bp which were used for the next round of scaffolding using the HiRise program. These scaffolds were subjected to gap-filling using all error-corrected SMRT reads and PBJelly program[6] which resulted in the final "PacBio-HiC" assembly. Supplementary Table 6 shows the comparative statistics of the previously published 454-assembly[21] and the current PacBio-HiC assembly.

Supplementary Table 6. Assembly statistics of the published 454- assembly[21] and the current PacBio-HiC assembly of the Japanese lamprey genome.

	**Published 454-assembly[21]**	**PacBio-HiC assembly**
Assembled genome size	1.03 Gb	1.07 Gb
Number of contigs	99,385	4,716
Contig N50 length	9.2 kb	1.6 Mb
Longest contig length	163.4 kb	14.7 Mb

Number of scaffolds	86,125	3,560
Scaffold N50 length	1.05 Mb	10.7 Mb
Longest scaffold length	12.8 Mb	29.7 Mb
Total gap length in the assembly	177.2 Mb	664.9 kb

Assembly quality and completeness

The quality of the PacBio-HiC assembly was determined by using 97,942 pairs of BAC-end reads generated previously from a Japanese lamprey BAC library (average insert size of ~100 kb) using DNA from the testis of the same male individual[21]. Alignment of the BAC end reads to the assembly using BLASTN identified 55,347 pairs that mapped uniquely to the assembly. Of these, 1,472 pairs mapped to different scaffolds (altogether 314 scaffolds) and were located >100 kb from the scaffold ends thus representing potential cases of Hi-C mis-assemblies. Of the 314 potentially mis-assembled scaffolds, 111 are shorter than 100 kb. Thus, there are no large-scale structural mis-assemblies in the genome assembly. Note that our reconstruction analysis is unlikely to be affected by such assembly errors (false joining), because we made synteny blocks by comparing Japanese lamprey scaffolds with the sea lamprey genome and several gnathostome genomes. The overall contiguity of the present assembly is better than that of the previous 454-assembly[21] of the Japanese lamprey (Supplementary Fig. 1b). To evaluate the completeness of the assembly, we used 978 metazoan genes from the OrthoDB v9 of the BUSCO v2.0[7]. We chose this set instead of 2,586 vertebrate genes as the BUSCO team has indicated that “vertebrata_odb9: excludes the sea lamprey (*Petromyzon marinus*) because of high sequence divergence, should thus be used for Gnathostomata as it is not ideal for Agnatha”. The assembly contained complete sequences for 83.5% of the metazoan genes whereas 3.5% of these genes were partial. The remaining 13% of the genes were missing from the assembly. We further evaluated the the assembly completeness using a dataset of TRINITY transcripts obtained by assembling RNA-seq reads from nine tissues of the Japanese lamprey (brain, accession number SRX2267405; eye, SRX2532946; heart,SRX2372719; intestine, SRX2267404; kidney,SRX2372734; muscle,SRX2372741; notochord,SRX2372747; ovary,SRX2372750; and testis,SRX2372773). The TRINITY transcripts were clustered at 97% identity and 80% coverage cut-offs using CD-HIT[8]. Approximately 137,000 of these CD-HIT-clustered transcripts that were ≥1 kb were aligned to the assembly using BLAT[9]. Around 93% of these transcripts mapped to the

assembly (with ≥90% coverage and ≥90% sequence identity) indicating that the assembly contained most of the genes.

Repetitive sequences

A *de novo* repeat library from the Japanese lamprey PacBio-HiC assembly was generated using RepeatModeler v1.0.10[10]. TEclass v2.1.3[11] was used to classify repetitive elements of unknown class. The repeat library was then merged with known Japanese lamprey repeats from the RepBase v22.05[12]. Cd-hit[8] was used with 94% as identity cut-off and 80% as coverage cut-off to cluster this combined repeat library. From each cluster, the longest repeat sequence was chosen as a representative in order to generate a non-redundant repeat library. These repeat sequences were further filtered using BLASTX[15, 16] with human proteins from RefSeq[13, 14] at an E-value cutoff of 10^{-20}. Repeat sequences with significant similarity to human proteins were removed from the repeat library. These typically represent repetitive domains found in protein sequences. The final Japanese lamprey genome-specific repeat library comprised of 1,832 repetitive elements. Prior to the genome annotation, repetitive sequences in the assembly were masked using this final repeat library.

Genome annotation

The MAKER pipeline v2.31.8[17] was used to perform the whole-genome annotation. First, the repeat library (see previous section) was used to identify and soft-mask repetitive regions within the genome assembly by using RepeatMasker v4.0[18]. The different types of repetitive sequences in the Japanese lamprey genome assembly are reported in Supplementary Table 7. It was found that around half (~50%) of the Japanese lamprey genome comprised repetitive sequences, which is more than twice the repeat content estimated in the previous 454-assembly[21] (21%).

Both evidence-based gene prediction as well as AUGUSTUS[19]-based *ab initio* gene prediction were performed on the Japanese lamprey PacBio-HiC genome assembly. During evidence-based annotation, a Cd-hit[8] clustered set of ~137,000 transcript sequences with length ≥1 kb from a transcriptome assembly generated from nine tissues in two previous studies[21, 22] were used. Other than this transcriptome dataset, a protein dataset of ~208,000 RefSeq[13] proteins from *Lethenteron*

camtschaticum, Petromyzon marinus, Strongylocentrotus purpuratus, Homo sapiens, Ciona intestinalis, Branchiostoma floridae, Danio rerio, Oryzias latipes, Xenopus tropicalis, Gallus gallus, Nematostella vectensis and Elasmobranchii were also used. In addition, 17,772 protein sequences previously predicted in the elephant shark 454-assembly[1] were also included. The transcript and protein sequence-based hint files in the GFF3 format were used as input during AUGUSTUS[19]-based *ab initio* gene prediction to improve the gene prediction process and to calculate the Annotation Edit Distance (AED) score[17]. An in-house Perl script was used to merge gene models from both the approaches which resulted in a total of 19,455 protein-coding genes with an AED score ≤0.5.

Supplementary Table 7. Repetitive sequences in the Japanese lamprey genome assembly.

Class	subclass	Count	Bp Masked	%masked
**DNA**		259,041	43,746,207	4.31%
	Academ-1	1,494	745,631	0.07%
	CMC-Chapaev-3	10,961	4,900,379	0.48%
	CMC-EnSpm	12,627	1,861,917	0.18%
	Crypton-H	1,833	129,865	0.01%
	Ginger	224	76,707	0.01%
	Novosib	33,868	3,943,917	0.39%
	TcMar-Mariner	234	59,430	0.01%
	TcMar-Tc1	29,905	6,567,530	0.65%
	TcMar-Tc2	732	106,085	0.01%
	TcMar-Tigger	19,703	6,296,099	0.62%
	Zator	36,868	5,487,510	0.54%
	Zisupton	70	3,831	0.00%
	hAT	206	162,769	0.02%
	hAT-Ac	4,982	1,350,149	0.13%
	hAT-Blackjack	876	173,507	0.02%
	hAT-Charlie	10,215	2,423,195	0.24%
	hAT-Tip100	13,649	3,301,048	0.33%
	hAT-hATm	241	53,920	0.01%
	**total**	**437,729**	**81,389,696**	**8.00%**
**LINE**		216,618	34,976,927	3.36%
	CR1-Zenon	2,716	455,982	0.04%
	I-Jockey	17,703	5,165,207	0.51%
	Jockey	5,226	1,104,742	0.11%

	L1	963	201,556	0.02%
	L1-Tx1	10,978	3,328,246	0.33%
	L2	104,706	18,502,645	1.82%
	Penelope	331,706	45,724,077	4.51%
	R2	65	61,745	0.01%
	R2-Hero	496	444,616	0.04%
	RTE-BovB	125,131	49,394,604	4.87%
	RTE-RTE	67,434	10,229,839	1.01%
	Rex-Babar	623	568,523	0.06%
	**total**	**884,365**	**170,158,709**	**17.00%**
**LTR**		92,239	20,236,356	1.99%
	Copia	733	230,562	0.02%
	ERV1	5,341	4,549,843	0.45%
	Gypsy	46,914	32,091,206	3.16%
	Gypsy-Cigr	3,631	8,814,466	0.87%
	Ngaro	61,801	9,389,176	0.93%
	Pao	1,231	1,157,919	0.11%
	**total**	**211,890**	**76,469,528**	**7.50%**
**RC**	Helitron	29,042	11,408,980	1.12%
**Retro**	*nosubclass*	164,722	19,547,864	1.93%
**SINE**	*nosubclass*	37,767	5,876,742	0.58%
	5S	3,461	289,036	0.03%
	Alu	2,709	423,815	0.04%
	B2	141	31,899	0.00%
	tRNA	215,137	37,438,553	3.69%
	tRNA-Core	49,742	8,725,907	0.86%
	tRNA-Deu	20,659	3,053,499	0.30%
	tRNA-Deu-L2	235	55,616	0.01%
	tRNA-RTE	8,688	843,209	0.08%
	tRNA-V	111,016	23,223,013	2.29%
		**411,788**	**74,084,547**	**7.30%**
**Unknown**		34,872	7,784,107	0.77%
**Total interspersed repeats**		**2,212,175**	**446,720,173**	44.03%
**Low_complexity**		36708	3,546,054	0.35%
**Satellite**		64656	10,023,864	0.99%
**Simple_repeat**		379524	46,713,954	4.60%
**rRNA**		260	106,311	0.01%

**snRNA**		149	89,456	0.01%
**Total**		2,693,472	507,199,812	49.99%

Supplementary Note 2. Annotation of orthologues and paralogues

We obtained orthologues and paralogues between gnathostome species from Ensembl gene trees[23] downloaded from ftp://ftp.ensembl.org/pub/release-75/emf/ensembl-compara/homologies/Compara.75.protein.nhx.emf.gz. Then we discarded genes that have lineage-specific small-scale duplications in order to avoid ambiguity in subsequent synteny analyses. Thus, duplicates were retained only if they were annotated in Ensembl as Vertebrata, Euteleostomi or Clupeocephala, which means they are likely to be ohnologues (i.e. paralogues created by WGD[24]). In addition, we discarded paralogues that were duplicated into more than 10 Vertebrata or Euteleostomi nodes. For orthologues between elephant shark and other gnathostomes, we chose reciprocal best BLASTP[16] (with the elephant shark genes treated as query sequences) matches as orthologues.

2.1 Orthologues between vertebrates and invertebrates

We performed BLASTP searches for all species pairs (with vertebrate genes as query sequences and invertebrate genes as subject sequences), and identified orthologues and paralogues using a method similar to previous studies[25, 26]. For example, we defined human orthologues to amphioxus genes as follows: first, we assigned each human gene to the best-matching amphioxus gene that had the largest bit-score; second, for each amphioxus gene, we chose the top four best-scoring human genes as orthologues.

2.2 Orthologues between cyclostomes and gnathostomes

Considering the possibility of cyclostome-specific WGD, we chose 1-to-2 orthologues between gnathostomes (human, chicken, spotted gar and elephant shark) and cyclostomes (Japanese lamprey and sea lamprey) as follows. We performed BLASTP searches for all species pairs (with cyclostome genes as query sequences and gnathostome genes as subject sequences) and assigned each lamprey gene to the best-scoring gene in individual gnathostome species (e.g. human). Then we chose the top two best-scoring cyclostome (e.g. Japanese lamprey) genes as orthologues. We repeated this for all species pairs between cyclostomes and gnathostomes.

2.3 Orthologues between sea lamprey and Japanese lamprey

We performed a BLASTP search between the sea lamprey genes (query) and the Japanese lamprey genes (subject) and defined reciprocal best hits as orthologues.

2.4 Cyclostome paralogues

Lamprey paralogues were annotated with stringent criteria in order to avoid paralogue identification errors caused by partially annotated genes. Specifically, a Japanese lamprey gene pair with BLASTP bit-score s was annotated as a paralogue pair if it satisfies the following three conditions. (1) The shorter gene does not have larger (than s) bit-scores to any amphioxus genes. (2) The shorter gene has a larger (than s) bit-score to the best-matching sea lamprey gene. These conditions ensure that the shorter gene diverged from its paralogue after divergence from its amphioxus orthologue but before divergence from its sea lamprey orthologue. (3) In order to exclude large gene families, we discarded the gene pair if it is not one of the top seven best-scoring BLASTP matches (excluding the self match) for either of the lamprey genes. (We retained seven paralogues for each gene because the number of ohnologues is at most eight if we assume three rounds of WGD.) We defined sea lamprey paralogues in the same way. In sum, we found 11,316 combinations of two Japanese lamprey genes (and 12,005 combinations of two sea lamprey genes) to be paralogous. These paralogues were used for reconstructing proto-cyclostome chromosomes.

2.5 Elephant shark paralogues

Elephant shark paralogues were annotated by using the same rules as in the Japanese lamprey paralogues described above, but replacing the Japanese lamprey genes and sea lamprey genes with the elephant shark genes and human genes, respectively. In addition, we discarded an elephant shark gene pair if it is not one of the top three best-scoring BLASTP matches for neither of the elephant shark genes. We found 9,914 combinations of two elephant shark genes to be paralogous.

Supplementary Note 3. Reconstruction analysis

3.1 Reconstruction of the proto-vertebrate genome

3.1.1 ***Number of proto-vertebrate chromosomes.*** To decide the optimal number of proto-vertebrate chromosomes, we computed the reconstruction significance (see Methods) for K=10, …, 20 as shown in Supplementary Table 8, and chose K=18 as the optimal reconstruction.

Supplementary Table 8. Reconstruction significance for *K*=10, …, 20 in the Japanese lamprey genome.

K	$\log(\mathbb{P}(X \geq x))$
10	-8363.48
11	-9003.43
12	-9438.88
13	-9705.24
14	-9958.95
15	-10079.3
16	-10182.1
17	-10508.8
18	-10767.3
19	-10371.5
20	-10249.1

3.1.2 *Comparison with previous proto-vertebrate genome reconstructions.* We compared our reconstruction with the previous reconstructions presented by Putnam et al.[27], Sacerdot et al.[28] and Simakov et al.[29] Supplementary Figure 3 shows that the 18 proto-vertebrate chromosomes are largely consistent among the three reconstructions. In addition, we compared our reconstruction with the scallop (*Chlamys farreri*) genome, as descried in the next subsection. The overall correspondence of reconstructed chromosomes is summarized in Supplementary Table 9. The comparison of our reconstruction with previous studies shows two major differences: (1) Pvc17 and Pvc18 corresponds to a single chromosome (CLG11) in the reconstructions by Putnam et al. or Simakov et al. and (2) Pvc8 and Pvc9 correspond to a single chromosome (chr14) in the reconstruction by Sacerdot et al. A comparison of conserved synteny between these proto-vertebrate chromosomes and the scallop genome shows that Pvc17, Pvc18, Pvc8 and Pvc9 correspond to individual scallop chromosomes — chromosomes 3, 13, 6 and 4, respectively. This observation supports our reconstruction with 18 chromosomes, suggesting that the four chromosomes in our reconstruction may have existed as distinct chromosomes in early invertebrate lineages.

Supplementary Table 9. Comparison of our reconstruction with previous reconstruction studies and the scallop genome.

This study	Putnam *et al*.	Sacerdot *et al*.	Simakov *et al*.	Scallop
Pvc1	CLG16	chr1	CLGB	chr15,18,19
Pvc2	CLG3	chr10	CLGD	chr1
Pvc3	CLG4	chr11	CLGJ	chr5,10
Pvc4	CLG5	chr12	CLGK	chr2
Pvc5	CLG10	chr17	CLGP	chr11
Pvc6	CLG9	chr16	CLGN	chr17
Pvc7	CLG8	chr15	CLGF	chr8
Pvc8	CLG6	chr14	CLGQ	chr6
Pvc9	CLG7	chr14	CLGI	chr4
Pvc10	CLG13	chr5	CLGE	chr7
Pvc11	CLG14	chr6	CLGO	chr2,16
Pvc12	CLG15	chr4	CLGH	chr6
Pvc13	CLG2	chr7	CLGC	chr12,14
Pvc14	CLG1	chr8	CLGL	chr5
Pvc15	CLG12	chr9	CLGM	chr15
Pvc16	CLG17	chr2	CLGG	chr9
Pvc17	CLG11	chr3	CLGA	chr3
Pvc18	CLG11	chr13	CLGA	chr13

3.1.3 *Comparison with invertebrate genomes.* Strong conservation of macrosynteny among invertebrate genomes has been reported in previous studies[27, 30, 31, 32, 33, 34]. Thus the individual proto-vertebrate chromosomes are likely to have distinct orthologue distributions in invertebrate lineages, which also serve as a validation of our reconstruction. Thus, we compared the proto-vertebrate genome with several invertebrate genomes: amphioxus (*Branchiostoma floridae*)[27], scallop (*Chlamys farreri*)[35, 36], freshwater snail (*Biomphalaria glabrata*)[37, 38], silkworm (*Bombyx mori*)[39], sea anemone (*Nematostella vectensis*)[30] and *Trichoplax adhaerens*[31].

In Supplementary Figure 4, we organized the amphioxus scaffolds into 18 groups that correspond to the proto-vertebrate chromosomes. For the freshwater snail genome, we obtained gene sequences from ref. [37] and the linkage map information from ref. [38]. Then, scaffolds were assigned to the linkage group with the largest number of markers. For the scallop genome, we obtained gene sequences, annotation and linkage marker information from refs. [35, 36]. Then we mapped markers to their best-matching scaffolds by using BLASTN as in ref. [34] and assigned scaffolds to the chromosome with the largest number of markers. For the sea anemone and

Trichoplax adhaerens genomes, we chose the largest (in terms of the number of genes) 50 scaffolds for plotting orthologues. The resulting figure (Supplementary Fig. 4) shows that reconstructed proto-vertebrate chromosomes (y-axis) have distinct orthologue distributions in invertebrate genomes, suggesting that they are likely to be separate chromosomes even in the early metazoan lineages.

3.1.4 ***Comparison between the Japanese lamprey and sea lamprey scaffolds.*** We also computed a reconstruction from the Japanese lamprey and sea lamprey scaffolds with at least five genes (without performing synteny segmentation) using the same parameter values as described in Methods (but numbers of post-WGD scaffolds were $C_1 = 227$ and $C_2 = 212$). Supplementary Figure 5 shows that (1) inter-chromosomal rearrangements are infrequent between the two lamprey lineages and (2) gene order is strongly conserved between the two lamprey genomes, often spanning entire chromosomes.

3.2 Reconstruction of the proto-gnathostome genome

3.2.1 ***Comparison with previous proto-gnathostome reconstructions.*** A previous reconstruction analysis[26] suggested some rearrangements (fusions or fissions) between the two WGD events, but it was not possible at that time to distinguish between chromosome fusions and fissions due to the lack of outgroup genomes. Our comparison between the proto-gnathostome and proto-vertebrate chromosomes indicated that nine large-scale rearrangements took place between the two WGD events (Supplementary Figs. 6 and 7), which is also consistent with a recent reconstruction[28]. For example, the overlap on the y-axis between Pvc2-3 (Supplementary Figs. 6 and 7) suggests a chromosome fusion between the two WGD events; similarly, chromosome fusions were inferred between Pvc3-4, 5-6, 4-6-7, 7-8-9, 8-9, 10-11, 13-14 and 14-15. The number of proto-gnathostome chromosomes was inferred to be 40 in ref. [26], which was smaller than 49 in our reconstruction because many segments were assigned to chrUn in ref. [26] and we found more proto-vertebrate chromosomes affected by fusion events between 1R and 2R. On the other hand, the proto-gnathostome chromosome number was 54 in ref. [28]; this difference is probably due to fusions involving multiple chromosomes in our reconstruction: e.g. fusions involving Pvc4-6-7 and Pvc7-8-9. Our analysis with the elephant shark genome suggests that such complex rearrangements took place between the two WGD events.

3.3 The proto-cyclostome genome was shaped by six-fold genome duplication.

To test if the observed peak of multiplicity at six can be explained by accumulation of chromosome-scale duplications, we calculated the probability that multiplicities of independently duplicating chromosomes converge toward a given ploidy level (see Methods). Supplementary Table 10 shows small probabilities of observing convergence of multiplicities through independent chromosome-scale duplications. Thus, it is unlikely that the proto-cyclostome genome was shaped by a series of independently occurring chromosome-scale duplications.

Supplementary Table 10. Small probabilities (P) that multiplicities of independently duplicating chromosomes converge to a given ploidy level (M).

Scenario	*K*	*Y*	*D*	*N*	*M*	*P*
A	17	103	13	1	6	0.0000000018
B	17	103	13	2	6	0.0000030304
C	17	103	13	4	6	0.0214209597
D	17	49	6	1	3	0.0000002044
E	17	49	6	2	3	0.0038115884
A	18	104	18	1	6	0.0000000480
B	18	105	17	2	6	0.0000371775
C	18	107	15	4	6	0.0487599825
D	18	50	8	1	3	0.0000011843
E	18	51	7	2	3	0.0067631372
A	5	30	0	1	6	0.0000421035
B	5	30	0	2	6	0.0003120318
C	5	30	0	4	6	0.0049925087
D	5	15	0	1	3	0.0009990010
E	5	15	0	2	3	0.0159840160

Supplementary Note 4. Evolution of proto-gnathostome and proto-cyclostome chromosomes

4.1 Previous arguments on the origin of microchromosomes in gnathostomes

The identification of microchromosomes in some early-diverging gnathostome lineages such as holocephalans (ratfish), chondrosteans (sturgeon) and holosteans (spotted gar) led to the suggestion that avian microchromosomes are remnants of microchromosomes that existed in

early diverging gnathostomes[40]. Recent studies tend to support this ancient-origins hypothesis: It was argued that many avian microchromosomes represent ancient chromosomes in the ancestral land vertebrate[54], and that many proto-gnathostome chromosomes are retained as microchromosomes in the chicken genome without inter-chromosomal rearrangements[26]. The strong conservation in gene content was confirmed in several studies[1, 26, 32, 41, 42, 43, 44], but little was known about the origin of chromosomal features that characterize avian microchromosomes (i.e. chromosome length, GC contents, etc). Comparative analysis between the spotted gar genome and chicken genome showed that the chromosomal features already presented in the common ancestor of bony-vertebrate[61], and our analysis with the chromosome-scale elephant shark genome showed that the origin dates back further to the proto-gnathostome, suggesting that those chromosomal features were likely to be associated with the subgenome fractionation after 2R.

4.2 Potential factors affecting the rearrangement rate of microchromosomes

Figure 5 shows that microchromosomes tend to have larger densities of ohnologues, which is consistent with the model that the rate of inter-chromosomal rearrangement is affected by dosage sensitive genes[45]. We examined other factors that might have affected the rate of inter-chromosomal rearrangement. In particular, we focused on genomic regulatory blocks (GRBs) and topologically associating domains (TADs), because such regulatory domains are under strong selective pressure in vertebrates and invertebrates[46].

We obtained GRB/TAD data from ref. [46] and analyzed their coverage and density as follows. For each proto-gnathostome chromosome, we calculated the total length of human segments that were assigned to the proto-gnathostome chromosome; next we calculated the coverage by GRBs/TADs (i.e. the fraction of bases in GRBs/TADs) and the density of GRB/TAD boundaries. We plotted these statistics (y-axis) against the proto-gnathostome chromosome size (x-axis) in Supplementary Figure 8. Unlike the densities of genes and ohnologues (Fig. 5), GRBs/TADs show no clear correlation with respect to the chromosome size. These data suggest that GRBs and TADs are not the major factors in the persistent conservation of microchromosomes.

4.3 Mechanisms of chromosome fusions between 1R and 2R

It was previously argued that early vertebrate lineages experienced two contrasting modes of genome structure evolution: i.e., some early vertebrate lineages had relatively stable (or slowing evolving) genome structure for a long evolutionary time, while other lineages had many chromosome fusion events in a relatively short period of evolutionary time[26, 47]. The proto-gnathostome lineage might have experienced a rapid transition from a phase of stable/slow karyotype evolution to a phase of frequent chromosome fusions. The mechanism is unknown, but karyotypic reversal (from acrocentric chromosomes to metacentric chromosomes) by Robertsonian fusions is observed in mammals[48], and a similar phenomenon might have occurred in the proto-gnathostome lineage.

4.4 Inferred evolutionary scenario and biased gene retention

The inferred evolutionary scenario presented in Figure 6 was produced as follows. First, we assigned distinct colours to the 18 proto-vertebrate chromosomes. The lengths of proto-vertebrate chromosomes were defined as the numbers of amphioxus genes assigned to the chromosomes. These chromosomes underwent first WGD (1R), resulting in the doubling of the proto-vertebrate genome. In the lineage leading to extant gnathostomes, this was followed by nine chromosome fusions and the second WGD event (2R). The coloured bars in the proto-gnathostome genome represent the number of genes retained in proto-gnathostome chromosomes: The lengths of the coloured bars were defined by $4x$, where x is the number of proto-gnathostome genes retained from the focal proto-vertebrate chromosome, and a proto-vertebrate gene was inferred to be retained on the proto-gnathostome chromosome if the gene has at least one orthologue in the human, mouse, dog, opossum, chicken, turkey, zebra finch, spotted gar or elephant shark segments assigned to the proto-gnathostome chromosome. By this definition, we expect that the entire proto-gnathostome chromosome is painted if the chromosome retains 1/4 of the genes on the ancestral proto-vertebrate chromosome. Most of the proto-gnathostome chromosomes have white regions because of biased gene loss, small-scale rearrangements, and lack of clear orthology relationship between amphioxus and gnathostome genes due to their sequence divergence for a long evolutionary time. In the cyclostome lineage, six proto-cyclostome chromosomes were shown for each proto-vertebrate chromosome as our analysis suggested genome triplication at the origin of cyclostomes post 1R. Where our

reconstruction produced fewer than six chromosomes, the remaining chromosomes out of the expected six are shown as hatched bars. Where our reconstruction produced more than six chromosomes, the extra chromosomes are not shown. However, the extra chromosomes were included in all other figures, including Figures 2 and 3, although they are very small. The lengths of coloured bars in the proto-cyclostome genome were defined by $6x$ (instead of $4x$ for proto-gnathostome chromosomes), because our analysis suggested that the proto-cyclostome genome experienced six-fold multiplication. Extant genomes were painted according to the colours assigned to the proto-vertebrate chromosomes. Stripes of multiple colours in the modern gnathostome genomes indicate that those regions derived from fusion chromosomes in the proto-gnathostome genome. Regions of the human genome shown in white likely correspond to regions poor in genes, such as centromeres and pericentromeric regions.

Figure 6 shows that duplicated fusion chromosomes in the proto-gnathostome genome exhibit biased gene retention, and that the chromosomes with higher rates of gene loss tend to be retained as microchromosomes in chicken and elephant shark. This observation suggests that microchromosomes and their distinctive chromosomal features (Fig. 5) were acquired as a result of biased fractionation of a subgenome after allo-tetraploidization, as discussed in refs [49, 50, 51]. Based on this observation, we classified the proto-gnathostome chromosomes into two subgenomes, which we call the L subgenome and the S subgenome as in ref. [52]. (The L subgenome is the longer one with a lower rate of gene loss, whereas the S subgenome is the shorter one with a higher rate of gene loss.) A pair of fusion chromosomes were compared with respect to the chromosome size (i.e. the total length of human segments mapped to the proto-gnathostome chromosome) shown in Figure 5, and the longest proto-gnathostome chromosome was classified as the L subgenome while the shortest proto-gnathostome was classified as the S subgenome. If a group of quadruple proto-gnathostome chromosomes were not affected by the fusions between 1R and 2R, we classified the longest two chromosomes as the L subgenome and the shortest two as the S subgenome. The resulting classification is shown in Figure 6e, where the proto-gnathostome chromosomes in the S subgenome are surrounded by thick line.

4.5 Functional biases between the two proto-gnathostome subgenomes

In order to investigate potential functional biases between the two subgenomes in the proto-gnathostome genome, we performed a gene ontology enrichment analysis. First, we classified the

human segments into two groups: (Group 1) segments mapped to the L subgenome and (Group 2) segments mapped to the S subgenome. Then, we performed a GO analysis between the human genes in Group 1 (L genes) and the genes in Group 2 (S genes) using GOrilla[53]. Supplementary Table 11 shows that the genes in Group 1 are overrepresented by metabolic process, cellular process, etc., and Supplementary Table 12 shows that the genes in Group 2 are overrepresented by cornification, keratinization, regulation of transcription, immune system process, etc. (FDR $q<10^{-4}$). In addition, we performed a GO analysis using PANTHER[54], and found that the genes in Group 2 are overrepresented by major histocompatibility complex protein, immunoglobulin receptor superfamily, and defense/immunity protein (PANTHER Protein Class, FDR $q<10^{-12}$) as shown in Supplementary Table 13.

Supplementary Table 11. GOs overrepresented in the human genes in the L subgenome.

ID	L genes	S genes	*p*-value	FDR	Description
GO:0008152	5395	1936	5.10E-14	7.78E-10	metabolic process
GO:0009987	8941	3452	1.27E-13	9.68E-10	cellular process
GO:0044237	4926	1756	3.79E-13	1.92E-09	cellular metabolic process
GO:0071704	5055	1821	3.61E-12	1.38E-08	organic substance metabolic process
GO:0044238	4805	1725	9.27E-12	2.83E-08	primary metabolic process
GO:0044281	1225	355	6.21E-11	1.58E-07	small molecule metabolic process
GO:0043436	700	180	4.92E-10	1.07E-06	oxoacid metabolic process
GO:0006082	711	184	5.58E-10	1.06E-06	organic acid metabolic process
GO:0030030	597	147	7.25E-10	1.23E-06	cell projection organization
GO:0006807	4470	1639	2.34E-08	3.57E-05	nitrogen compound metabolic process
GO:0019752	632	168	2.71E-08	3.76E-05	carboxylic acid metabolic process

Supplementary Table 12. GOs overrepresented in the human genes in the S subgenome.

ID	L genes	S genes	*p*-value	FDR	Description
GO:0070268	23	82	5.04E-25	7.68E-21	cornification
GO:0031424	55	100	9.70E-20	7.39E-16	keratinization
GO:0006355	1934	1020	8.58E-12	4.36E-08	regulation of transcription, DNA-templated
GO:1903506	1969	1035	1.04E-11	3.96E-08	regulation of nucleic acid-templated transcription
GO:2001141	1974	1036	1.35E-11	4.11E-08	regulation of RNA biosynthetic process
GO:0010468	2571	1301	2.49E-11	6.33E-08	regulation of gene expression
GO:0051252	2137	1106	2.87E-11	6.24E-08	regulation of RNA metabolic process
GO:0010556	2315	1175	2.22E-10	4.23E-07	regulation of macromolecule biosynthetic process
GO:2000112	2236	1138	3.01E-10	5.11E-07	regulation of cellular macromolecule biosynthetic process
GO:0007156	62	75	4.18E-10	6.37E-07	homophilic cell adhesion via plasma membrane adhesion molecules
GO:0019219	2354	1179	2.35E-09	3.26E-06	regulation of nucleobase-containing compound metabolic process
GO:0031326	2439	1215	3.14E-09	3.99E-06	regulation of cellular biosynthetic process
GO:0045087	226	170	4.26E-09	4.99E-06	innate immune response
GO:0002376	1177	639	6.60E-09	7.18E-06	immune system process
GO:0050911	143	120	1.35E-08	1.37E-05	detection of chemical stimulus involved in sensory perception of smell
GO:0009889	2496	1231	1.42E-08	1.35E-05	regulation of biosynthetic process
GO:0006955	474	294	3.29E-08	2.95E-05	immune response
GO:0019730	45	55	7.03E-08	5.95E-05	antimicrobial humoral response

Supplementary Table 13. PANTHER Protein Classes overrepresented in the human genes in the L and S subgenomes.

ID	L genes	S genes	*p*-value	FDR	Description
PC00149	1	13	2.30E-06	5.61E-05	major histocompatibility complex protein
PC00124	24	84	1.02E-23	1.99E-21	immunoglobulin receptor superfamily
PC00090	139	156	2.82E-15	2.75E-13	defense/immunity protein
PC00248	200	190	1.28E-13	8.33E-12	C2H2 zinc finger transcription factor
PC00244	251	214	4.37E-12	2.13E-10	zinc finger transcription factor
PC00218	546	365	1.76E-09	5.72E-08	DNA-binding transcription factor
PC00264	599	395	1.12E-09	4.35E-08	gene-specific transcriptional regulator
PC00262	976	305	7.20E-09	2.01E-07	metabolite interconversion enzyme

Supplementary Note 5. Gene tree analysis

5.1 Classification of duplication timing

Lineage-specific WGDs are expected to have created pairs of duplicated chromosomes that have large numbers of lineage-specific paralogues. In order to confirm this, we plotted paralogues among proto-gnathostome and proto-cyclostome chromosomes and classified them into vertebrate paralogues (i.e. duplicated in the common ancestral vertebrate), cyclostome-specific paralogues and gnathostome-specific paralogues as described below.

Human paralogues annotated as Vertebrata in Ensembl BioMart were classified as vertebrate paralogues (blue dots), and human paralogues annotated as Euteleostomi were classified as gnathostome-specific paralogues (red dots). Supplementary Figure 9 shows the distribution of vertebrate and gnathostome-specific paralogues mapped onto the proto-gnathostome genome.

For classifying lamprey paralogues into vertebrate and cyclostome-specific paralogues, we performed a comprehensive gene tree analysis by inserting lamprey genes into Ensembl gene trees. The numbers of annotated paralogues are shown in Supplementary Table 14, in which annotation methods A, B, C, D and E indicate (A) gene-tree-based annotation with WAG matrix, (B) robust annotation that was unchanged by replacing WAG/JTT/LG matrices, (C) annotation of low GC-content paralogues with WAG matrix, (D) annotation based on gene trees inferred with RAxML instead of using Ensembl gene trees, and (E) paralogue annotation for sea lamprey genes instead of Japanese lamprey genes. Method details are described below.

Supplementary Table 14. Numbers of lamprey paralogue pairs reannotated by gene tree analysis.

Annotation method	Paralogue pairs annotated as		Total
	vertebrate-specific	cyclostome-specific	
A	3646	1842	5488
B	2728	1578	4306
C	1798	1184	2982
D	2353	1990	4343
E	3537	1922	5459

A. Tree-based paralogue annotation**.** For each Ensembl gene tree[23], we chose orthologous lamprey genes and inserted them into the tree using RAxML-EPA[55] as follows.

Step 1: For each lamprey gene, we chose the best matching BLASTP hit among the human, chicken and spotted gar genes in Ensembl gene trees, and assigned the lamprey gene to the gene tree.

Step 2: For each gene tree, we chose lamprey genes that were assigned to the tree as described in Step 1. Then we built an alignment profile from the multiple alignment (precomputed by Ensembl) of all amino acid sequences in the tree, and performed profile alignment with the lamprey genes using HMMER (http://hmmer.org/).

Step 3: In order to reduce the computation time, we retained genes from *H. sapiens, G. gallus, L. chalumnae, L. oculatus, P. marinus, C. intestinalis, C. savingnyi, D. melanogaster* and *C. elegans*, and deleted the remaining genes from the tree. Then, we inserted the lamprey genes into the tree using RAxML-EPA[55] with the WAG amino acid substitution matrix.

Step 4: We parsed the output tree from RAxML-EPA, and annotated lamprey paralogues according to the tree topology. Specifically, lamprey paralogues were annotated as cyclostome-specific if the duplication node is ancestral to at least one sea lamprey gene, at least one Japanese lamprey gene, no gnathostome genes and no outgroup invertebrate genes; on the other hand, they were annotated as vertebrate-specific if the duplication node is ancestral to at least one gnathostome gene and no outgroup genes. If more than two lamprey genes from the same species were inserted into the same node, those multifurcating genes were excluded from the list of paralogues.

Step 5: We expected that Japanese lamprey and sea lamprey genes would cluster together if the gene tree was reliable; alternatively, lamprey genes can be isolated in the tree due to unreliable gene annotations, alignments or gene tree inference. Therefore, lamprey genes were excluded from the paralogue list if they were isolated in the tree. This step reduced the number of positionally isolated paralogues and tandemly duplicated paralogues, which are unlikely to be ohnologues.

The resulting paralogue distribution is shown in Supplementary Figure 10.

B. Confirmation by robustly annotated paralogues**.** We repeated the above RAxML-EPA analysis by replacing the amino acid substitution matrix (i.e. JTT and LG instead of WAG) for checking consistency. Then we retained gene pairs that were annotated as vertebrate paralogues (or as cyclostome paralogues) consistently in the three replicates with different matrices. We observed that a small number of short sequences were inserted into different branches when the substitution matrix was replaced, but as a whole, we confirmed that the resulting paralogue annotation was largely consistent. The paralogue distribution is shown in Supplementary Figure 11.

C. Confirmation with low-GC paralogues. In addition, we excluded genes with high GC content in order to see if the true phylogenetic signals can be recovered by using only low-GC sequences. We sorted all genes by GC content and removed the third of the genes with the highest GC content. Then the paralogues between the remaining low-GC genes were re-annotated using RAxML-EPA with the WAG matrix. The low-GC paralogues had a similar distribution pattern (Supplementary Fig. 12), suggesting that our observation was not substantially biased by high-GC genes.

D. Confirmation by gene tree inference with RAxML without using Ensembl gene trees. We excluded *P. marinus* in Step 3, and inferred gene trees from the alignments in Step 2 (using RAxML with the WAG substitution matrix, instead of just inserting lamprey genes into Ensembl gene trees using RAxML-EPA). To exclude tandem duplications and partially annotated genes, we retained only one-to-one orthologues between Japanese lamprey and sea lamprey (i.e. a pair of lamprey genes are one-to-one orthologues if the two lamprey genes are only descendants of their common ancestor node). The result is shown in Supplementary Figure 13.

E. Confirmation with sea lamprey paralogues. Finally, we performed the same analyses with the sea lamprey genes and observed similar paralogue distributions. The annotation of the sea

lamprey paralogues was done by using RAxML-EPA with the WAG matrix (method A), and is shown in Supplementary Figure 14.

We considered that gnathostome and cyclostome lineage-specific polyploidization events must have created sets of newly duplicated chromosomes that share large numbers of lineage-specific ohnologues. Our analysis (Supplementary Figs. 9–14) showed that (a) a pair of Hox-bearing proto-cyclostome chromosomes (Hox β and ε) share a large number of cyclostome-specific paralogues (see also Fig. 15a) as previously suggested[56] and (b) duplicated fusion chromosomes in the proto-gnathostome genome tend to share large numbers of gnathostome-specific paralogues, providing support to the hypothesis that the proto-cyclostome and proto-gnathostome genomes experienced lineage-specific polyploidizations after the shared 1R event.

On the other hand, these paralogue data (Supplementary Figs. 9–14) consistently show the presence of large numbers of vertebrate paralogues among majority of homoeologous proto-cyclostome chromosomes. Supplementary Figure 15 summarizes the distributions of orthologues and paralogues among selected proto-gnathostome and proto-cyclostome chromosomes. Supplementary Figure 15a shows the Hox-bearing chromosomes duplicated from Pvc1. If the cyclostome-specific genome triplication occurred long after 1R and after rediploidization, we expect to observe 2×3 structure of paralogy relationship among the six homoeologous proto-cyclostome chromosomes. Supplementary Figure 15 shows the lack of expected 2×3 structure (in terms of the number/density of paralogues or enrichment of cyclostome-specific paralogues), which suggests that the cyclostome-specific genome triplication occurred shortly after 1R and before rediploidization. In particular, a large number of cyclostome-specific paralogues are found between proto-cyclostome chromosomes with Hox clusters β and ε (Fig. 15a), which may suggest delayed rediploidization between the two chromosomes.

Supplementary Figures

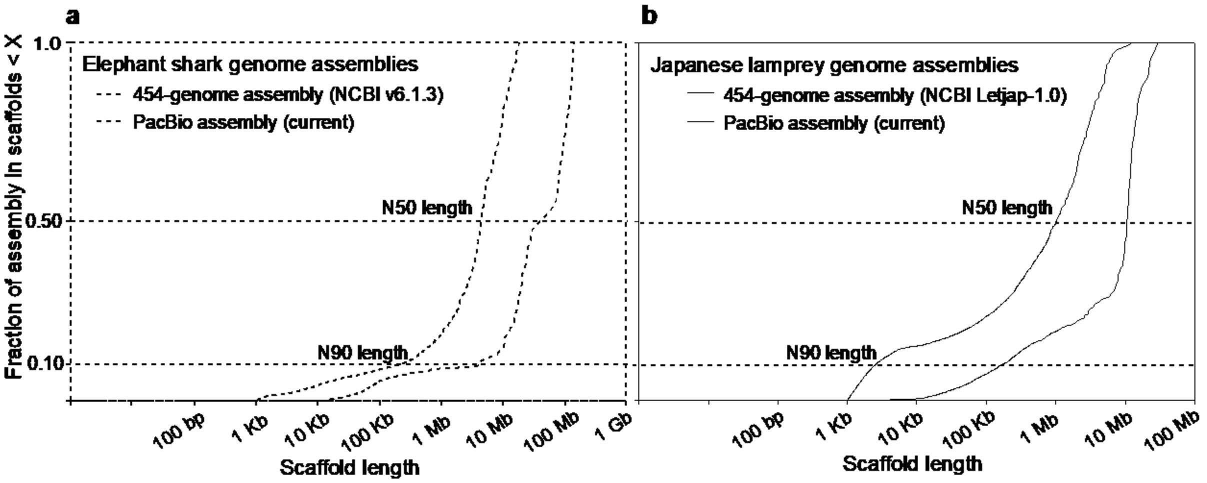

Supplementary Fig. 1: The comparison of contiguity between previous and current genome assemblies of (a) elephant shark and (b) Japanese lamprey. The curves display fraction of total length of the assembly present on scaffolds of given length or smaller.

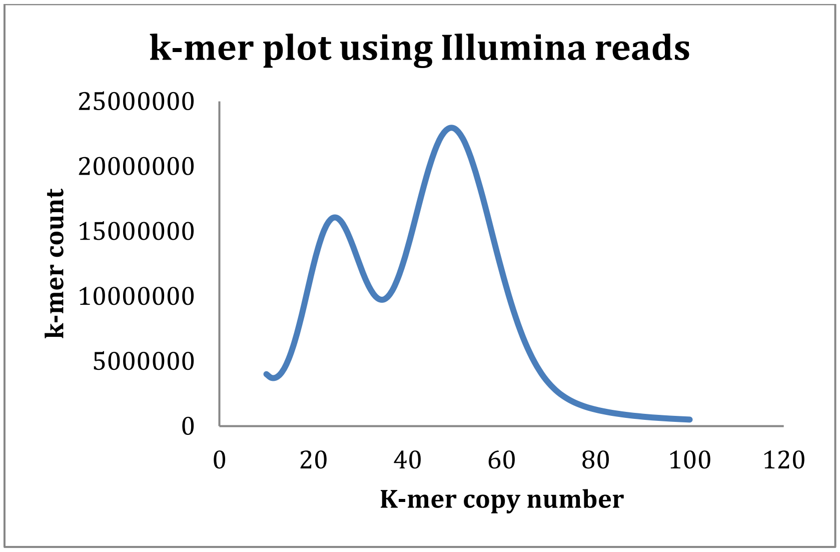

Supplementary Fig. 2. k-mer plot showing distribution of KCN at 25-mer for the Japanese lamprey genome. For better visualization of KCN distribution, only values between 11 and 100 are shown in the graph.

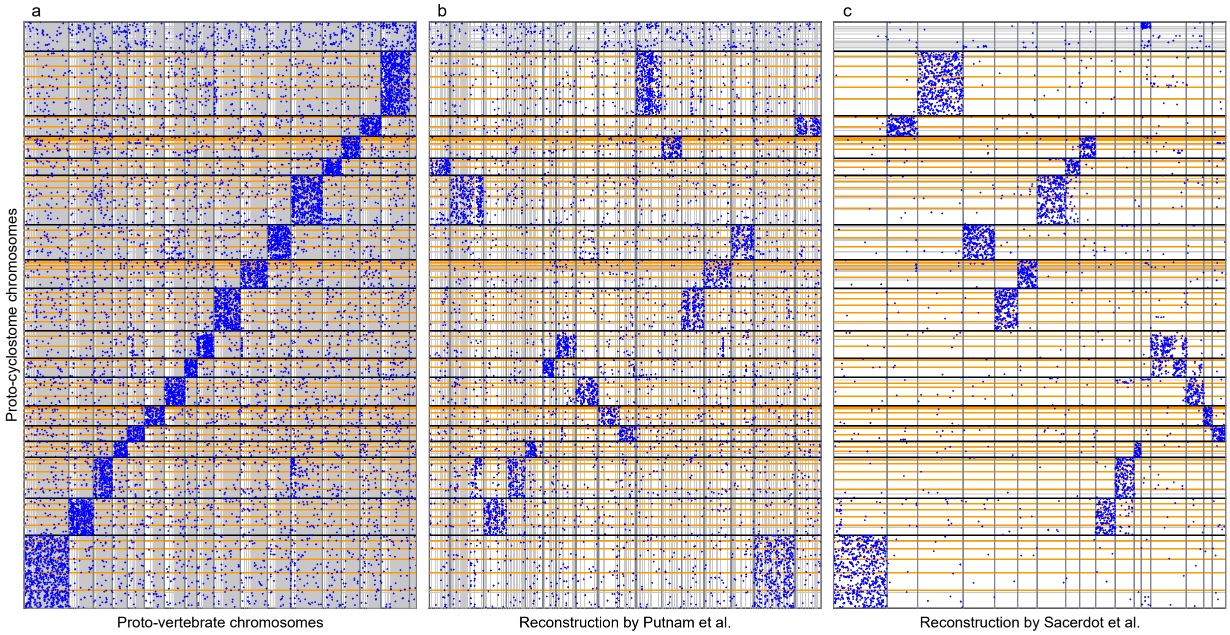

Supplementary Fig. 3. Comparison with previous reconstructions. We validated our reconstruction of proto-vertebrate chromosomes by comparing with the previous reconstructions in ref. [27] and ref. [28]. (**a)** Orthologues between amphioxus and Japanese lamprey. The x-axis shows our reconstruction of proto-vertebrate chromosomes represented by amphioxus scaffolds (Pvc1–Pvc18 from left to right), and the y-axis shows the proto-cyclostome chromosomes represented by Japanese lamprey scaffolds. Black and orange lines indicate boundaries of proto-vertebrate and proto-cyclostome chromosomes, respectively. Thin lines indicate boundaries of lamprey segments or amphioxus scaffolds. (**b)** Comparison between the proto-cyclostome chromosomes and the reconstruction by Putnam et al.[27] The x-axis shows the ancient chordate linkage groups represented by amphioxus scaffolds (CLG1–CLG17 from left to right). (**c**) Comparison between the proto-cyclostome chromosomes and the reconstruction by Sacerdot et al.[28] The x-axis shows the olfactores chromosomes represented by human genes. These comparisons show that our reconstruction is largely consistent with previous reconstructions.

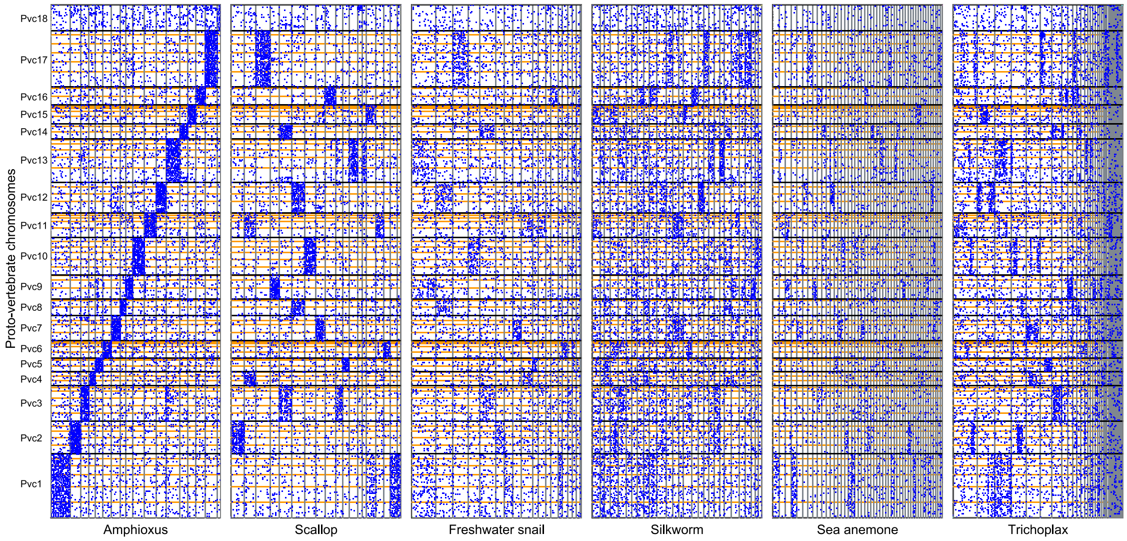

Supplementary Fig. 4. Validation of the proto-vertebrate reconstruction by comparison with invertebrate genomes. Japanese lamprey segments that were mapped to the proto-vertebrate/-cyclostome chromosomes are shown on the y-axis. Black and orange horizontal lines indicate boundaries of proto-vertebrate chromosomes and proto-cyclostome chromosomes, respectively. The proto-vertebrate chromosomes were compared with invertebrate genomes (x-axes). Blue dots represent orthologues between Japanese lamprey and invertebrate species. Vertical lines indicate boundaries of chromosomes, linkage groups or scaffolds. The distinct orthologue distributions for individual proto-vertebrate chromosomes (y-axis, Pvc1–Pvc18 from bottom to top) suggest that the reconstructed proto-vertebrate chromosomes indeed existed as separate chromosomes in the proto-vertebrate lineage.

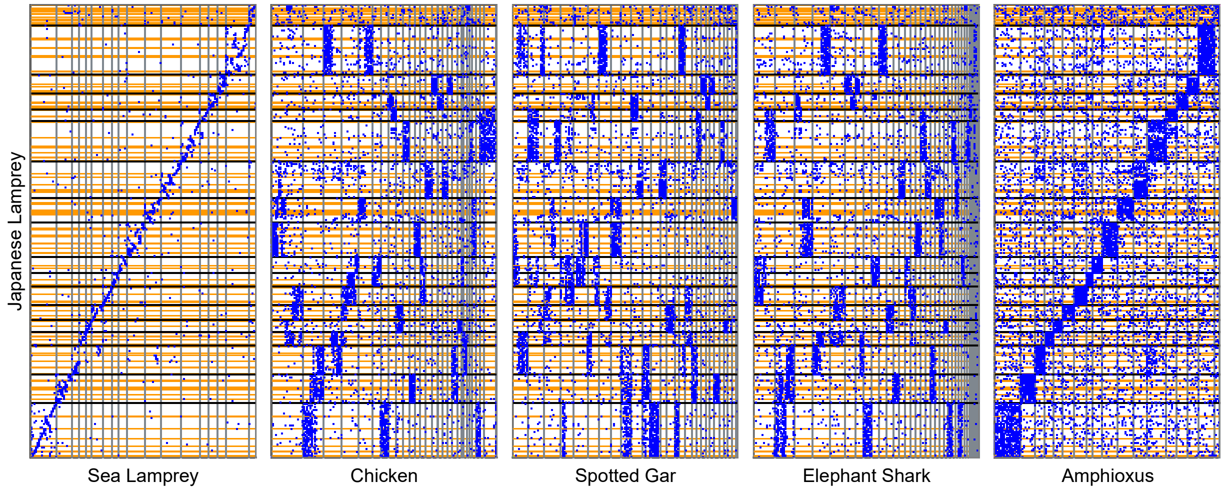

Supplementary Fig. 5. Low rate of inter-chromosomal rearrangement in lamprey. Orthologues were plotted between Japanese lamprey scaffolds (y-axis) and sea lamprey scaffolds, chicken chromosomes (chr1–Z and two LGs, i.e. LGE22C19W28_E50C23 and LGE64), spotted gar LGs (LG1–29), elephant shark scaffolds (sorted by the number of genes and the largest 50 scaffolds are shown) and amphioxus scaffolds (grouped into Pvc1–Pvc18). Japanese lamprey and sea lamprey scaffolds were arranged in 18 groups that roughly correspond to the proto-vertebrate chromosomes. Mostly diagonal distribution of orthologues indicates that inter-chromosomal rearrangements were infrequent between the two lamprey lineages. However, some lamprey scaffolds experienced inter-chromosomal rearrangements, which are visible as off-diagonal clusters of dots in the leftmost orthologue plot. The rightmost plot shows that the rate of inter-chromosomal rearrangement in lamprey has been remarkably low for ~500 million years: there are only a small number (~20) of rearrangements, which are visible as off-diagonal horizontal bands in this plot. These off-diagonal bands are absent in Figure 2, because lamprey scaffolds were partitioned into blocks of conserved synteny in our reconstruction analysis.

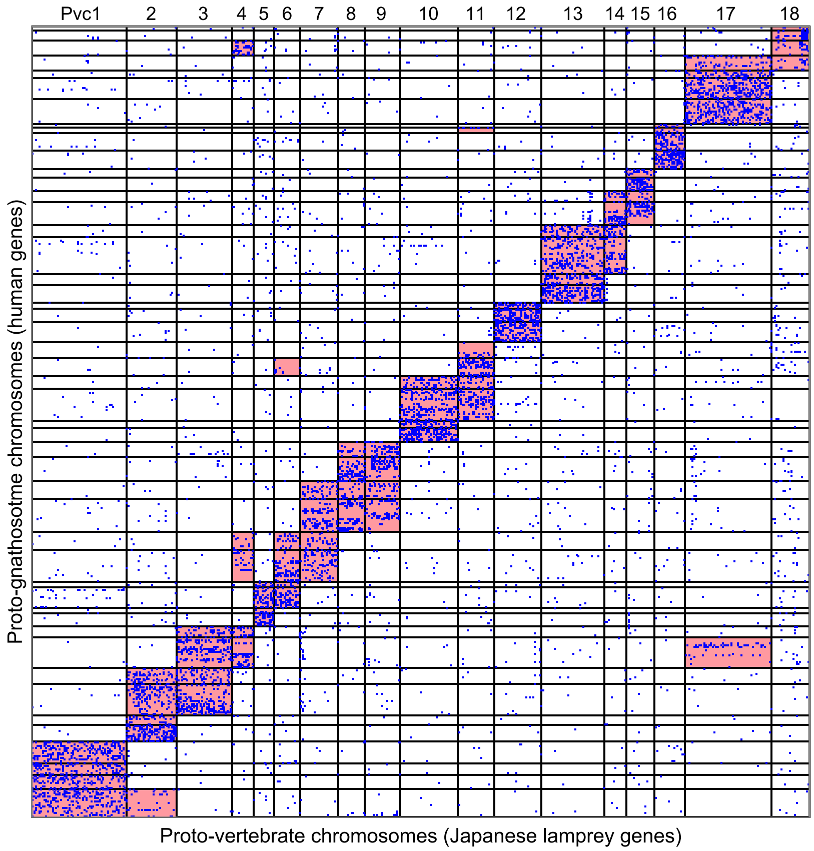

Supplementary Fig. 6. Detection of inter-2R fusion events. We compared the proto-vertebrate and proto-gnathostome chromosomes in order to identify rearrangements between the two genomes. The x-axe shows the proto-vertebrate chromosomes (Pvc1 to 18 from left to right) represented by Japanese lamprey genes. The y-axis shows the proto-gnathostome chromosomes represented by human genes. Red rectangles indicate chromosome pairs with significantly large numbers of orthologues ($P < 10^{-2}$, calculated using the Poisson distribution assuming that orthologue dots are randomly distributed). We inferred inter-2R chromosome fusion events if multiple proto-vertebrate chromosomes share two syntenic proto-gnathostome chromosomes with significantly large numbers of orthologues. For example, Pvc2 and Pvc3 share two syntenic proto-gnathostome chromosomes, which suggests a fusion before the second WGD in the gnathostome lineage. In the same way, inter-2R fusion events were inferred with respect to nine proto-vertebrate chromosome groups: namely, Pvc2-3, 3-4, 5-6, 4-6-7, 7-8-9, 8-9, 10-11, 13-14 and 14-15. These large-scale rearrangements were not found in the proto-cyclostome genome, suggesting that the proto-cyclostome lineage diverged from the proto-gnathostome lineage before these inter-2R fusion events.

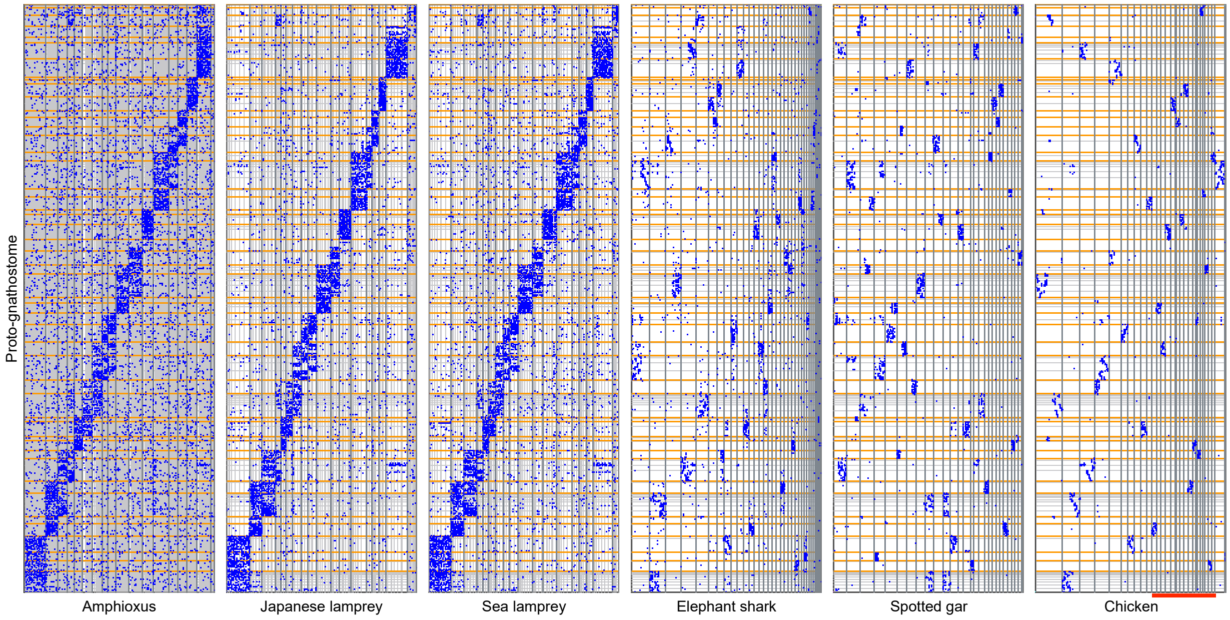

Supplementary Fig. 7. Comparison of the proto-gnathostome genome with the amphioxus, lamprey and gnathostome genomes. The proto-gnathostome chromosomes represented by human segments (y-axis) were compared with amphioxus, Japanese lamprey, sea lamprey, elephant shark, spotted gar and chicken (x-axis). Orange and grey horizontal lines indicate boundaries of proto-gnathostome chromosomes and human segments, respectively. For the amphioxus and two lampreys, thick vertical lines correspond to boundaries of proto-vertebrate chromosomes; thin vertical lines indicate boundaries of amphioxus scaffolds or lamprey segments. For the gnathostome genomes on the x-axis, vertical lines indicate boundaries of elephant shark scaffolds, spotted gar linkage groups or chicken chromosomes. This figure shows that (1) proto-vertebrate chromosomes (shown in the amphioxus panel) are orthologous to four proto-gnathostome chromosomes, supporting the 2R hypothesis; (2) nine cases exist in which two out of four duplicated proto-gnathostome chromosomes are orthologous to multiple proto-vertebrate chromosomes, suggesting chromosome fusions between the two WGD events in the proto-gnathostome lineage; (3) these fusion events are not shared with the lamprey genomes; and (4) many of the proto-gnathostome chromosomes are still retained as single chromosomes in the modern gnathostome genomes; in particular, many proto-gnathostome chromosomes are retained entirely as chicken microchromosomes (i.e. chr11–32 in this figure, indicated by the red line).

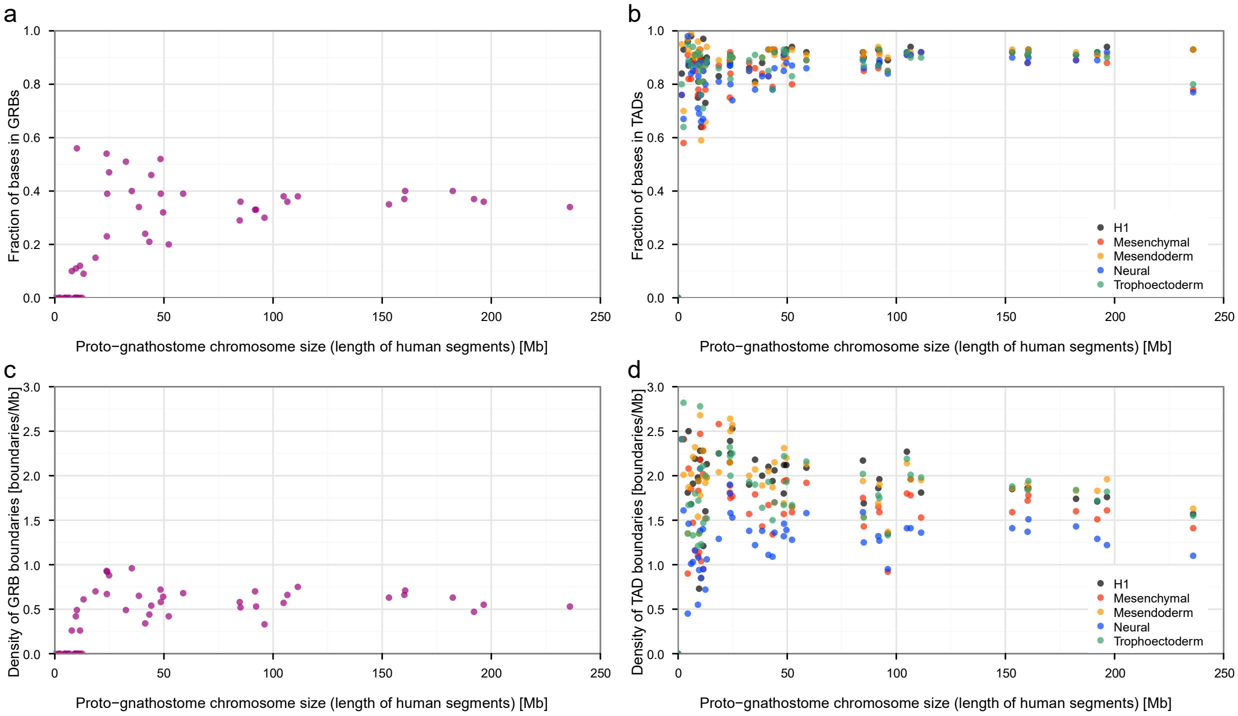

Supplementary Fig. 8. The proto-gnathostome chromosome length shows no clear association with genomic regulatory blocks (GRBs) or topologically associating domains (TADs). The x-axis shows the length of proto-gnathostome chromosomes (i.e. the total length of human segments assigned to the chromosome). The chromosome lengths do not correlate with the fraction of bases in GRBs (Panel a) or TADs (Panel b), nor with the density of GRB (Panel c) or TAD (Panel d) boundaries.

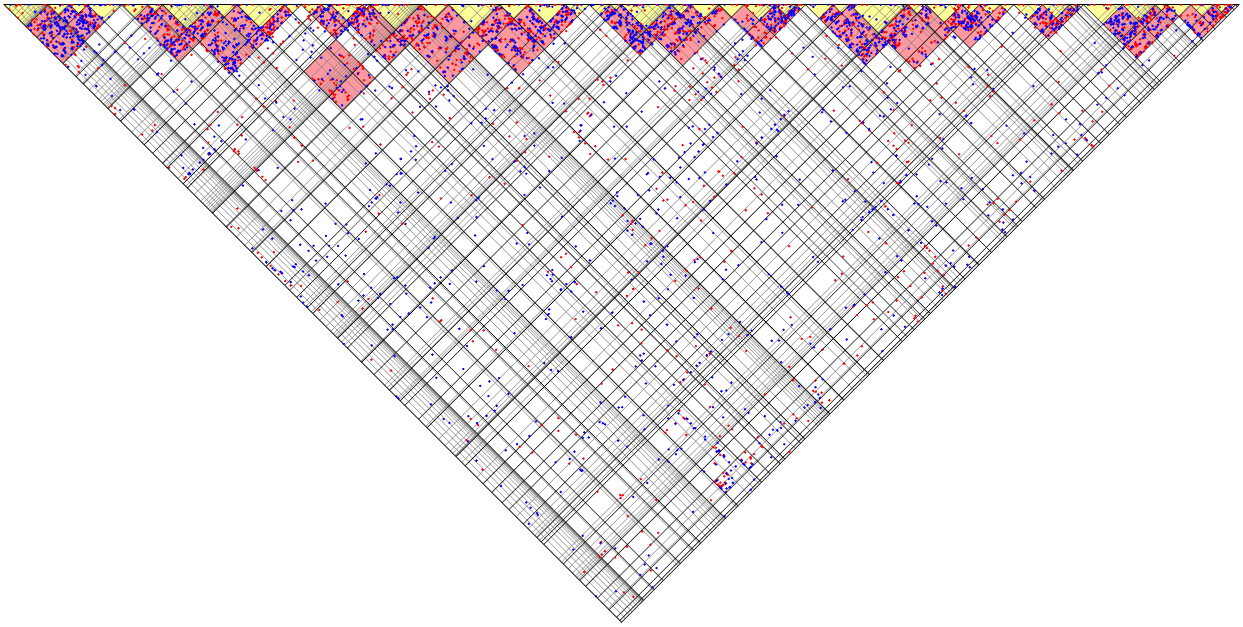

Supplementary Fig. 9. Paralogue distribution in the proto-gnathostome genome. The triangular scatterplot shows paralogues in the proto-gnathostome chromosomes. The proto-gnathostome chromosomes are represented by human segments as described in Fig. 4, with thick and thin lines indicating boundaries of proto-gnathostome chromosomes and human segments, respectively. Red regions indicate gene pairs between duplicated chromosomes, and yellow regions indicate gene pairs within the same proto-gnathostome chromosomes. We annotated duplicated chromosomes based on the set partitioning analysis (Fig. 4) and inter-2R fusion analysis (Fig. 6). Blue and red dots indicate vertebrate paralogues (i.e. annotated as Vertebrata) and gnathostome-specific paralogues (i.e. annotated as Euteleostomi) in Ensembl. Selected parts of this plot are presented in Fig. 14.

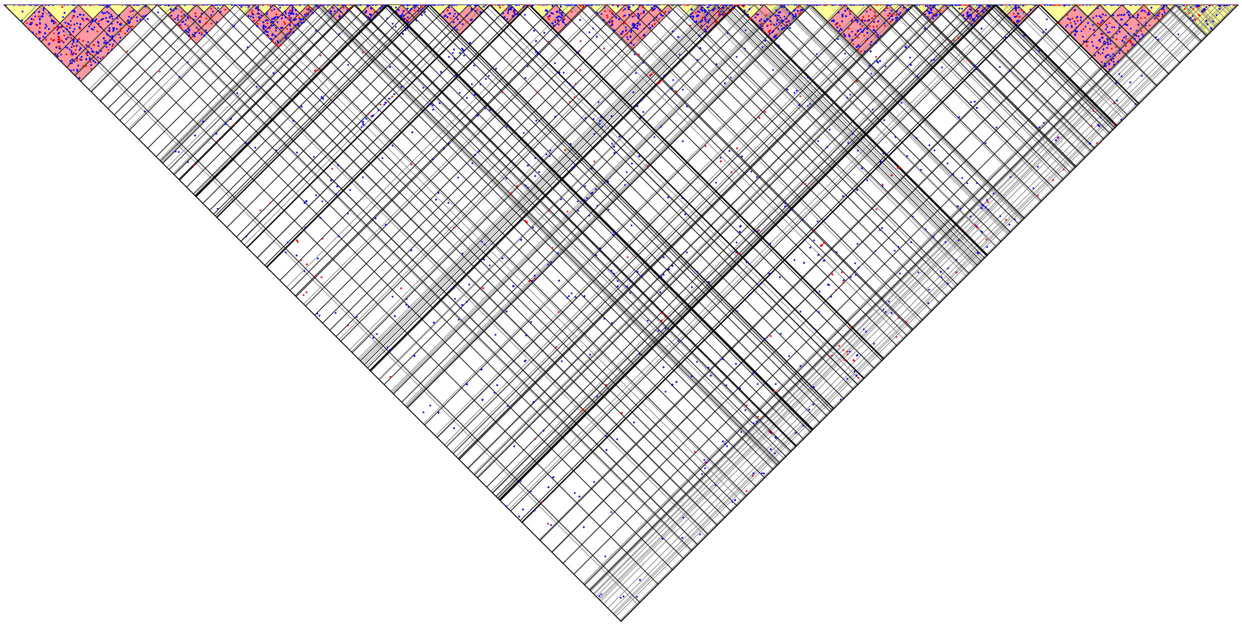

Supplementary Fig. 10. Distribution of Japanese lamprey paralogues annotated by using RAxML-EPA with the WAG matrix. The triangular scatterplot shows paralogues in the proto-cyclostome chromosomes. The proto-cyclostome chromosomes are represented by Japanese lamprey segments, with thick and thin lines indicating boundaries of proto-cyclostome chromosomes and Japanese lamprey segments, respectively. Red regions indicate gene pairs between duplicated chromosomes, and yellow regions indicate gene pairs within the same proto-cyclostome chromosomes. Blue and red dots indicate vertebrate paralogues and cyclostome-specific paralogues annotated by using RAxML-EPA with the WAG matrix. Selected parts of this plot are presented in Fig. 3.

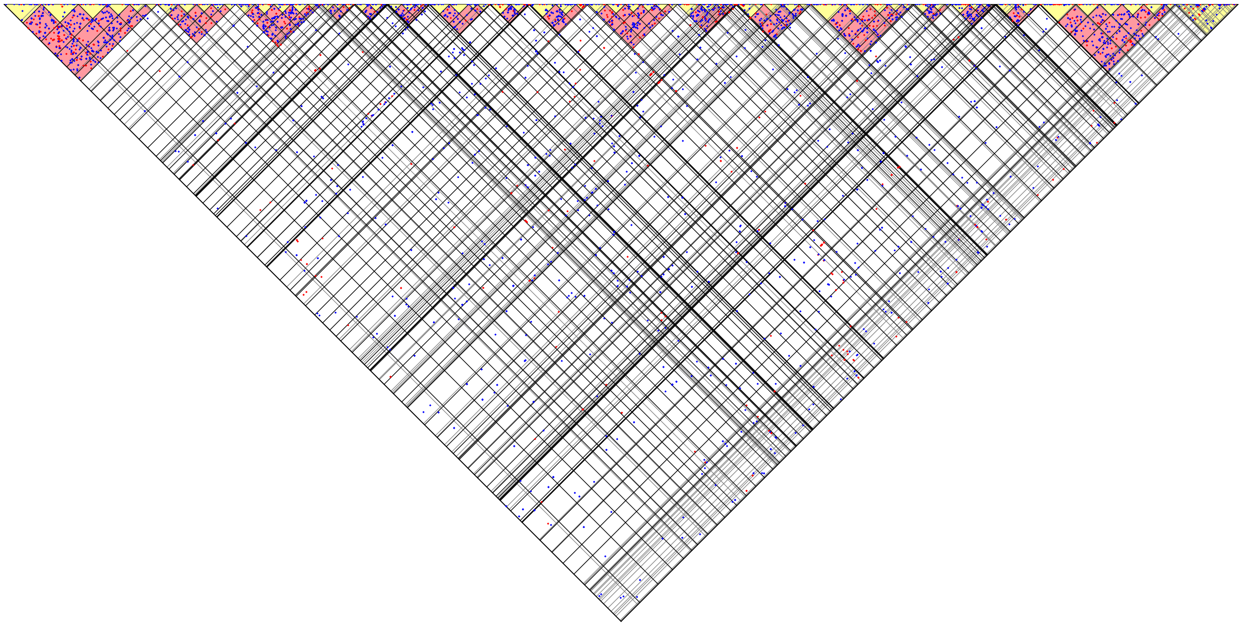

Supplementary Fig. 11. Distribution of Japanese lamprey paralogues annotated with the WAG, JTT and LG matrices. Japanese lamprey paralogues were annotated as vertebrate or cyclostome-specific paralogues using RAxML-EPA with three substitution matrices: WAG, JTT and LG. This plot shows paralogues that were annotated consistently as vertebrate or cyclostome-specific in all the three replications.

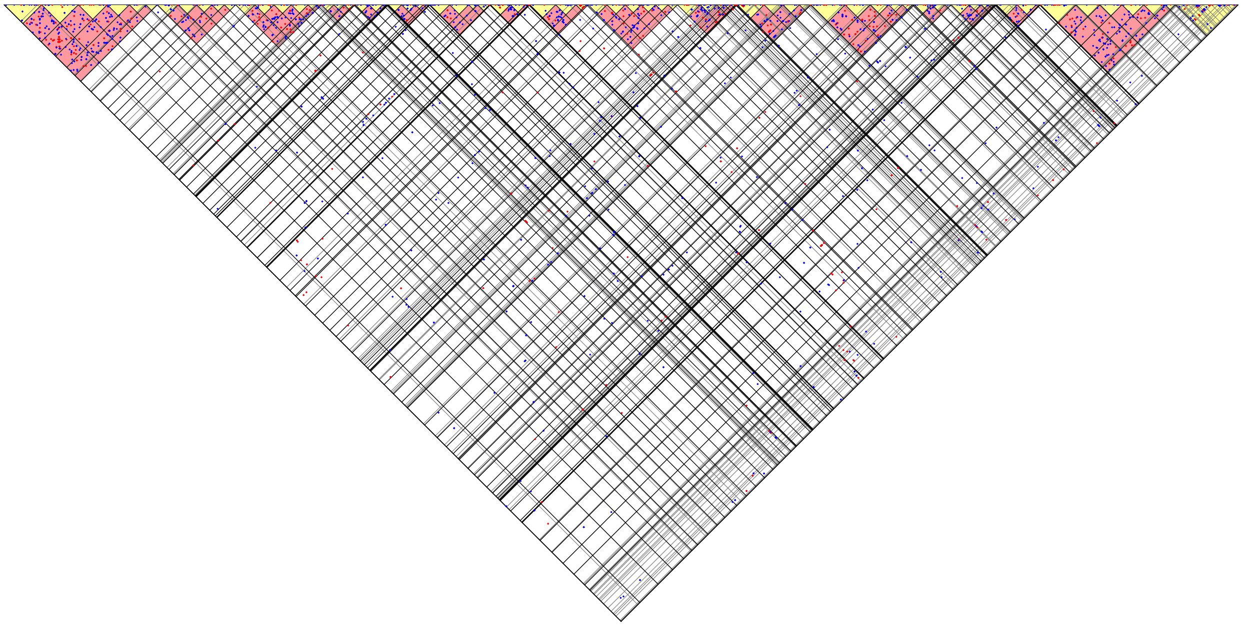

Supplementary Fig. 12. Distribution of Japanese lamprey paralogues with low GC-content. One third of high-GC genes were removed and the paralogue pairs between the remaining low-GC genes were re-annotated using RAxML-EPA with the WAG matrix.

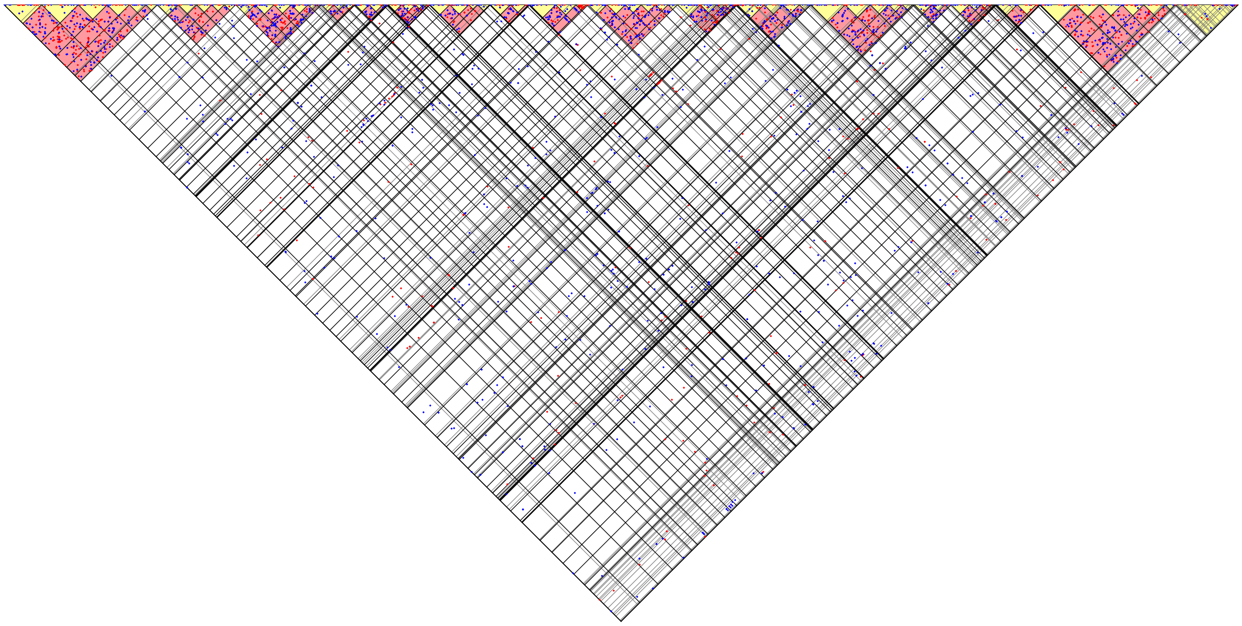

Supplementary Fig. 13. Distribution of Japanese lamprey paralogues annotated using gene trees inferred by RAxML. Instead of inserting lamprey genes into existing Ensembl gene trees using RAxML-EPA, gene trees were inferred using RAxML with the WAG matrix, and one-to-one orthologues between Japanese lamprey and sea lamprey were retained for paralogue annotation.

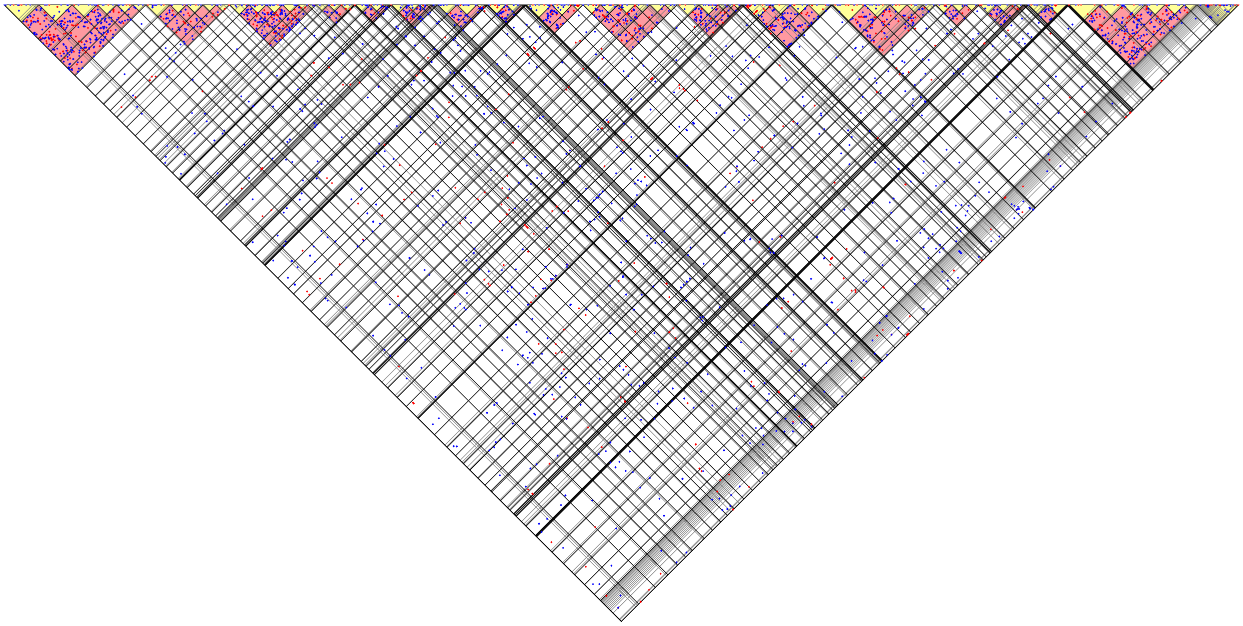

Supplementary Fig. 14. Distribution of sea lamprey paralogues annotated by using RAxML-EPA with the WAG matrix. Sea lamprey paralogues were shown instead of Japanese lamprey paralogues in Fig. 9.

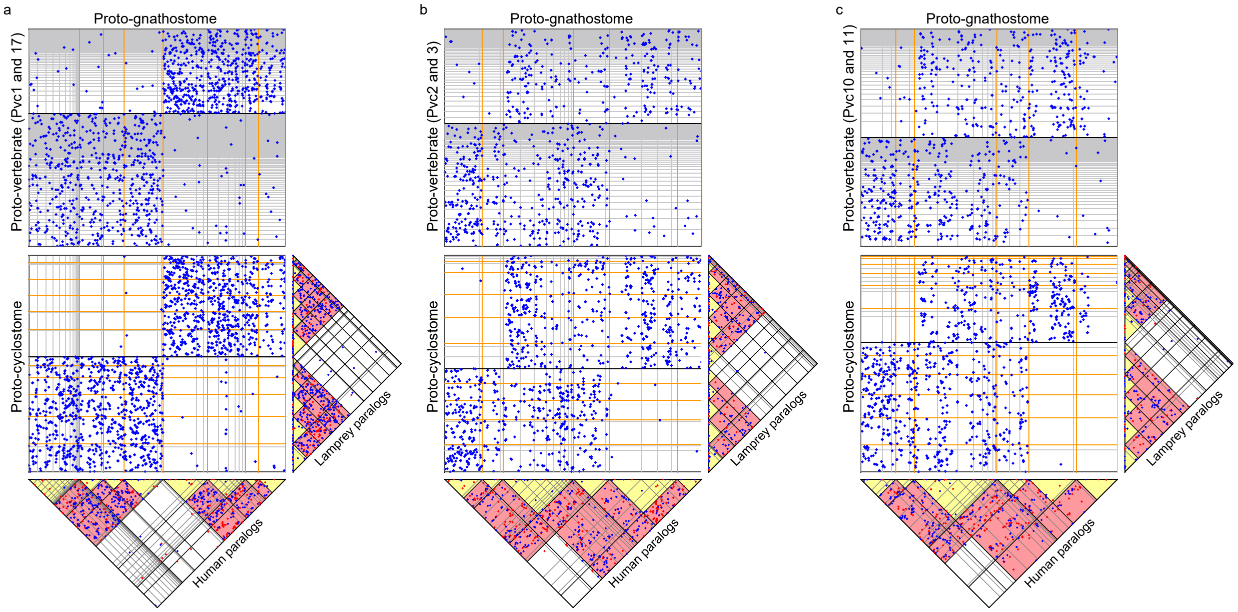

Supplementary Fig. 15. Evidence for the timing of gnathostome-cyclostome divergence. The rectangular scatter plots show orthologues among proto-gnathostome (x-axis), proto-vertebrate (y-axis, top) and proto-cyclostome (y-axis, bottom) chromosomes, which are represented respectively by human segments, amphioxus scaffolds and Japanese lamprey segments. Thin grey lines indicate boundaries of amphioxus scaffolds or lamprey/human segments. Thick black and orange lines respectively indicate boundaries of proto-vertebrate and proto-cyclostome/proto-gnathostome chromosomes. The triangular plots show paralogues classified into two groups: blue dots indicate vertebrate paralogues, and red dots indicate either gnathostome- or cyclostome-specific paralogues. Red regions indicate gene pairs between duplicated chromosomes, and yellow regions indicate intra-chromosome gene pairs within post-WGD chromosomes. (a) Comparison for Hox-bearing Pvc1 (bottom) and Pvc17 (top). The bottom six proto-cyclostome chromosomes are Hox-bearing chromosomes duplicated from Pvc1 (and have Hox clusters ε, α, β, γ, δ and ζ from bottom to top). The orthologue plot shows lack of one-to-one orthology relationship between proto-gnathostome and proto-cyclostome chromosomes, suggesting early divergence of the two lineages (shortly after the first WGD). In Panels b and c, the middle two out of six proto-gnathostome chromosomes have experienced chromosome fusion between the two WGD events, and thus they are syntenic to two proto-vertebrate chromosomes. Those fusions are not shared with the proto-cyclostome lineage,

suggesting that the fusions and subsequent second WGD occurred after the gnathostome-cyclostome split.

Supplementary References

1. Venkatesh B, *et al.* Elephant shark genome provides unique insights into gnathostome evolution. *Nature* **505**, 174-179 (2014).
2. Chin CS, *et al.* Phased diploid genome assembly with single-molecule real-time sequencing. *Nat Methods* **13**, 1050-1054 (2016).
3. Chin CS, *et al.* Nonhybrid, finished microbial genome assemblies from long-read SMRT sequencing data. *Nat Methods* **10**, 563-569 (2013).
4. Walker BJ, *et al.* Pilon: an integrated tool for comprehensive microbial variant detection and genome assembly improvement. *PLOS ONE* **9**, e112963 (2014).
5. Putnam NH, *et al.* Chromosome-scale shotgun assembly using an in vitro method for long-range linkage. *Genome Res* **26**, 342-350 (2016).
6. English AC, *et al.* Mind the gap: upgrading genomes with Pacific Biosciences RS long-read sequencing technology. *PLoS One* **7**, e47768 (2012).
7. Simao FA, Waterhouse RM, Ioannidis P, Kriventseva EV, Zdobnov EM. BUSCO: assessing genome assembly and annotation completeness with single-copy orthologs. *Bioinformatics* **31**, 3210-3212 (2015).
8. Li W, Godzik A. Cd-hit: a fast program for clustering and comparing large sets of protein or nucleotide sequences. *Bioinformatics* **22**, 1658-1659 (2006).
9. Kent WJ. BLAT--the BLAST-like alignment tool. *Genome Res* **12**, 656-664 (2002).
10. Smit A, Hubley R. RepeatModeler Open-1.0. <http://www.repeatmasker.org>. (2008-2015).
11. Abrusan G, Grundmann N, DeMester L, Makalowski W. TEclass--a tool for automated classification of unknown eukaryotic transposable elements. *Bioinformatics* **25**, 1329-1330 (2009).
12. Kapitonov VV, Jurka J. A universal classification of eukaryotic transposable elements implemented in Repbase. *Nat Rev Genet* **9**, 411-412; author reply 414 (2008).
13. O'Leary NA, *et al.* Reference sequence (RefSeq) database at NCBI: current status, taxonomic expansion, and functional annotation. *Nucleic Acids Res* **44**, D733-745 (2016).
14. Pruitt KD, Tatusova T, Maglott DR. NCBI reference sequences (RefSeq): a curated non-redundant sequence database of genomes, transcripts and proteins. *Nucleic Acids Res* **35**, D61-65 (2007).
15. Altschul SF, Gish W, Miller W, Myers EW, Lipman DJ. Basic local alignment search tool. *J Mol Biol* **215**, 403-410 (1990).
16. Camacho C, *et al.* BLAST+: architecture and applications. *BMC Bioinformatics* **10**, 421 (2009).
17. Cantarel BL, *et al.* MAKER: an easy-to-use annotation pipeline designed for emerging model organism genomes. *Genome Res* **18**, 188-196 (2008).
18. Smit A, Hubley R, Green P. RepeatMasker Open-4.0. <http://www.repeatmasker.org>. (2013-2015).
19. Stanke M, Diekhans M, Baertsch R, Haussler D. Using native and syntenically mapped cDNA alignments to improve de novo gene finding. *Bioinformatics* **24**, 637-644 (2008).
20. Marcais G, Kingsford C. A fast, lock-free approach for efficient parallel counting of occurrences of k-mers. *Bioinformatics* **27**, 764-770 (2011).
21. Mehta TK, *et al.* Evidence for at least six Hox clusters in the Japanese lamprey (*Lethenteron japonicum*). *Proc Natl Acad Sci U S A* **110**, 16044-16049 (2013).

22. Zhang H, *et al.* Lampreys, the jawless vertebrates, contain only two ParaHox gene clusters. *Proc Natl Acad Sci U S A* **114**, 9146-9151 (2017).
23. Vilella AJ, Severin J, Ureta-Vidal A, Heng L, Durbin R, Birney E. EnsemblCompara GeneTrees: Complete, duplication-aware phylogenetic trees in vertebrates. *Genome Res* **19**, 327-335 (2009).
24. Wolfe K. Robustness—it's not where you think it is. *Nat Genet* **25**, 3-4 (2000).
25. Dehal P, Boore JL. Two rounds of whole genome duplication in the ancestral vertebrate. *PLoS Biol* **3**, e314 (2005).
26. Nakatani Y, Takeda H, Kohara Y, Morishita S. Reconstruction of the vertebrate ancestral genome reveals dynamic genome reorganization in early vertebrates. *Genome Res* **17**, 1254-1265 (2007).
27. Putnam NH, *et al.* The amphioxus genome and the evolution of the chordate karyotype. *Nature* **453**, 1064-1071 (2008).
28. Sacerdot C, Louis A, Bon C, Berthelot C, Roest Crollius H. Chromosome evolution at the origin of the ancestral vertebrate genome. *Genome Biol* **19**, 166 (2018).
29. Simakov O, *et al.* Deeply conserved synteny resolves early events in vertebrate evolution. *Nat Ecol Evol* **4**, 820-830 (2020).
30. Putnam NH, *et al.* Sea anemone genome reveals ancestral eumetazoan gene repertoire and genomic organization. *Science* **317**, 86-94 (2007).
31. Srivastava M, *et al.* The *Trichoplax* genome and the nature of placozoans. *Nature* **454**, 955-960 (2008).
32. Louis A, Roest Crollius H, Robinson-Rechavi M. How much does the amphioxus genome represent the ancestor of chordates? *Brief Func Genomics* **11**, 89-95 (2012).
33. Simakov O, *et al.* Insights into bilaterian evolution from three spiralian genomes. *Nature* **493**, 526-531 (2013).
34. Wang S, *et al.* Scallop genome provides insights into evolution of bilaterian karyotype and development. *Nat Ecol Evol* **1**, 0120 (2017).
35. Li Y, *et al.* Scallop genome reveals molecular adaptations to semi-sessile life and neurotoxins. *Nat Commun* **8**, 1721 (2017).
36. Guo H, *et al.* Estimating realized heritability for growth in Zhikong scallop (*Chlamys farreri*) using genome-wide complex trait analysis. *Aquaculture* **497**, 103-108 (2018).
37. Adema CM, *et al.* Whole genome analysis of a schistosomiasis-transmitting freshwater snail. *Nat Commun* **8**, 15451 (2017).
38. Tennessen JA, Bollmann SR, Blouin MS. A targeted capture linkage map anchors the genome of the Schistosomiasis vector snail, *Biomphalaria glabrata*. *G3 (Bethesda)* **7**, 2353 (2017).
39. The International Silkworm Genome Consortium. The genome of a lepidopteran model insect, the silkworm *Bombyx mori*. *Insect Biochem Mol Biol* **38**, 1036-1045 (2008).
40. Ohno S, Muramoto J, Stenius C, Christian L, Kittrell WA, Atkin NB. Microchromosomes in holocephalian, chondrostean and holostean fishes. *Chromosoma* **26**, 35-40 (1969).
41. Burt DW. Origin and evolution of avian microchromosomes. *Cytogenet Genome Res* **96**, 97-112 (2002).
42. Voss SR, *et al.* Origin of amphibian and avian chromosomes by fission, fusion, and retention of ancestral chromosomes. *Genome Res* **21**, 1306-1312 (2011).

43. Uno Y*, et al.* Inference of the protokaryotypes of amniotes and tetrapods and the evolutionary processes of microchromosomes from comparative gene mapping. *PLOS ONE* **7**, e53027 (2012).
44. Braasch I, *et al.* The spotted gar genome illuminates vertebrate evolution and facilitates human-teleost comparisons. *Nat Genet* **48**, 427-437 (2016).
45. Lv J, Havlak P, Putnam NH. Constraints on genes shape long-term conservation of macro-synteny in metazoan genomes. *BMC Bioinformatics* **12**, S11 (2011).
46. Harmston N, Ing-Simmons E, Tan G, Perry M, Merkenschlager M, Lenhard B. Topologically associating domains are ancient features that coincide with Metazoan clusters of extreme noncoding conservation. *Nat Commun* **8**, (2017).
47. Nakatani Y, McLysaght A. Genomes as documents of evolutionary history: a probabilistic macrosynteny model for the reconstruction of ancestral genomes. *Bioinformatics* **33**, i369-i378 (2017).
48. Pardo-Manuel de Villena F, Sapienza C. Female meiosis drives karyotypic evolution in mammals. *Genetics* **159**, 1179-1189 (2001).
49. Garsmeur O, Schnable JC, Almeida A, Jourda C, D’Hont A, Freeling M. Two evolutionarily distinct classes of paleopolyploidy. *Mol Biol Evol* **31**, 448-454 (2014).
50. Wendel JF, Lisch D, Hu G, Mason AS. The long and short of doubling down: polyploidy, epigenetics, and the temporal dynamics of genome fractionation. *Curr Opin Genet Dev* **49**, 1-7 (2018).
51. Cheng F, Wu J, Cai X, Liang J, Freeling M, Wang X. Gene retention, fractionation and subgenome differences in polyploid plants. *Nat Plants* **4**, 258-268 (2018).
52. Session AM, *et al.* Genome evolution in the allotetraploid frog Xenopus laevis. *Nature* **538**, 336-343 (2016).
53. Eden E, Navon R, Steinfeld I, Lipson D, Yakhini Z. GOrilla: a tool for discovery and visualization of enriched GO terms in ranked gene lists. *BMC Bioinformatics* **10**, 48-48 (2009).
54. Mi H, Muruganujan A, Ebert D, Huang X, Thomas PD. PANTHER version 14: more genomes, a new PANTHER GO-slim and improvements in enrichment analysis tools. *Nucleic Acids Res* **47**, D419-D426 (2019).
55. Berger SA, Krompass D, Stamatakis A. Performance, accuracy, and web server for evolutionary placement of short sequence reads under maximum likelihood. *Syst Biol* **60**, 291-302 (2011).
56. Smith JJ*, et al.* The sea lamprey germline genome provides insights into programmed genome rearrangement and vertebrate evolution. *Nat Genet* **50**, 270-277 (2018).
