# Supplementary material for: Reconstruction of proto-vertebrate, proto-cyclostome and proto-gnathostome genomes provides new insights into early vertebrate evolution
Source: Nat Commun. 2021 Jul 23;12:4489. doi: 10.1038/s41467-021-24573-z (PMC8302630; doi:10.1038/s41467-021-24573-z)
Supplement: Supplementary file 3 — Description of Additional Supplementary Files [file 41467_2021_24573_MOESM3_ESM.pdf]

Description of Additional Supplementary Files

File Name: Supplementary Data 1
Description: The dataset shows the definition of the protovertebrate chromosomes, the proto-cyclostome chromosomes and the proto-gnathostome chromosomes with statistics including the numbers of genes and paralogues. It also includes the orthologues and paralogues that were used in the reconstructions, the definition of segments, and the linkage group information for the scallop and freshwater snail genomes

File Name: Supplementary Movie 1
Description: The movie shows exploration of alternative scenarios during the reconstruction of proto-cyclostome chromosomes in Figure 3. In our reconstruction, we expect that paralogues (dots in c) are enriched between duplicated chromosomes (red region in c) and are depleted within individual chromosomes (yellow triangles in c) in the proto-cyclostome genome. Based on this expectation, we wish to find the optimal reconstruction with the most significant enrichment of dots within the red region. In this movie, we generated 3600 alternative combinations of lamprey segments, all of which were less significant than the optimal one presented in Figure 3 (as we can see from the presence of many dots in the yellow triangles). During this enumeration, we have no prior assumption with respect to the number of duplication events. We conclude that the proto-cyclostome genome was shaped by six-fold duplication of the proto-vertebrate genome because nine out of the 18 proto-vertebrate chromosomes were duplicated into six proto-cyclostome chromosomes in our reconstruction, and the majority of the Japanese lamprey genes were mapped to these six-fold duplicated proto-cyclostome chromosomes (Panel d).
