# Supplementary material for: Reconstruction of proto-vertebrate, proto-cyclostome and proto-gnathostome genomes provides new insights into early vertebrate evolution
Source: Nat Commun. 2021 Jul 23;12:4489. doi: 10.1038/s41467-021-24573-z (PMC8302630; doi:10.1038/s41467-021-24573-z)
Supplement: Supplementary file 4 — Supplementary Data 1 [file 41467_2021_24573_MOESM4_ESM.zip › SuppData1/ChromosomeStatistics.pdf]

The tables in the following pages show the numbers of genes and paralogs on the reconstructed proto-cyclostome and proto-gnathostome chromosomes.

Table 1. Statistics for the proto-cyclostome chromosomes. Each line shows (1) proto-vertebrate chromosome name (Pvc), (2) number of amphioxus genes mapped to the Pvc, (3) proto-cyclostome chromosome name (Pcc), (4) number of Japanese lamprey genes mapped to the Pcc, (5) number of sea lamprey genes mapped to the Pcc, and (6) number of amphioxus genes that are mapped to the Pvc and are orthologous to lamprey genes mapped to the Pcc.

Table 2. Statistics for the proto-gnathostome chromosomes. Each line shows (1) proto-gnathostome chromosome name (Pgc), (2) number of human genes mapped to the Pgc, (3) number of chicken genes mapped to the Pgc, (4) number of spotted gar genes mapped to the Pgc, (5) number of elephant shark genes mapped to the Pgc, and (6) number of amphioxus genes that are mapped to the Pvc as visualized in Figure 6e.

Table 3. Numbers of paralogs between proto-cyclostome chromosomes. The numbers in each cell indicate Japanese lamprey paralogs and sea lamprey paralogs between two proto-cyclostome chromosomes. Empty cells indicate no paralogs. (Please magnify this file to see the numbers.)

Table 4. Numbers of paralogs between proto-gnathostome chromosomes. The numbers in each cell indicate human paralogs, chicken paralogs, spotted gar paralogs and elephant shark paralogs between two proto-gnathostome chromosomes. Empty cells indicate no paralogs. (Please magnify this file to see the numbers.)

Table 1. Statistics for the proto-cyclostome chromosomes.

Proto-vertebrate	Amphioxus genes	Proto-cyclostome	Japanese lamprey genes	Sea lamprey genes	Orthologous amphioxus genes
Pvc1	1445	Pcc1A	515	473	304
Pvc1	1445	Pcc1B	502	452	283
Pvc1	1445	Pcc1C	397	344	250
Pvc1	1445	Pcc1D	303	266	177
Pvc1	1445	Pcc1E	226	217	154
Pvc1	1445	Pcc1F	158	179	121
Pvc2	891	Pcc2A	287	326	174
Pvc2	891	Pcc2B	252	228	167
Pvc2	891	Pcc2C	206	225	154
Pvc2	891	Pcc2D	172	177	115
Pvc2	891	Pcc2E	155	184	78
Pvc3	686	Pcc3A	264	265	99
Pvc3	686	Pcc3B	261	234	96
Pvc3	686	Pcc3C	237	220	89
Pvc3	686	Pcc3D	231	212	88
Pvc3	686	Pcc3E	120	131	52
Pvc3	686	Pcc3F	62	114	47
Pvc4	473	Pcc4A	171	169	89
Pvc4	473	Pcc4B	143	171	67
Pvc4	473	Pcc4C	127	177	66
Pvc4	473	Pcc4D	18	0	1
Pvc4	473	Pcc4E	0	16	1
Pvc4	473	Pcc4F	4	4	0
Pvc5	525	Pcc5A	190	201	112
Pvc5	525	Pcc5B	189	175	96
Pvc5	525	Pcc5C	50	44	20
Pvc5	525	Pcc5D	0	38	11
Pvc5	525	Pcc5E	10	22	6
Pvc5	525	Pcc5F	7	8	2
Pvc5	525	Pcc5G	9	0	1
Pvc6	586	Pcc6A	182	212	105
Pvc6	586	Pcc6B	188	171	86
Pvc6	586	Pcc6C	108	101	55
Pvc6	586	Pcc6D	46	56	33
Pvc6	586	Pcc6E	36	26	17
Pvc6	586	Pcc6F	0	61	9
Pvc6	586	Pcc6G	10	0	0
Pvc7	707	Pcc7A	271	266	173
Pvc7	707	Pcc7B	260	271	155
Pvc7	707	Pcc7C	124	112	66
Pvc7	707	Pcc7D	162	25	43
Pvc7	707	Pcc7E	11	0	2
Pvc7	707	Pcc7F	0	10	2
Pvc8	420	Pcc8A	276	252	130
Pvc8	420	Pcc8B	207	212	100
Pvc8	420	Pcc8C	40	32	10
Pvc8	420	Pcc8D	15	15	4
Pvc8	420	Pcc8E	0	11	3
Pvc8	420	Pcc8F	2	4	0
Pvc9	563	Pcc9A	355	344	174
Pvc9	563	Pcc9B	277	282	159
Pvc9	563	Pcc9C	145	145	30
Pvc9	563	Pcc9D	23	0	3
Pvc10	962	Pcc10A	257	240	151
Pvc10	962	Pcc10B	252	240	148
Pvc10	962	Pcc10C	218	228	129
Pvc10	962	Pcc10D	196	205	120
Pvc10	962	Pcc10E	172	202	115
Pvc10	962	Pcc10F	128	170	90
Pvc11	844	Pcc11A	314	296	167
Pvc11	844	Pcc11B	225	261	126
Pvc11	844	Pcc11C	107	132	74
Pvc11	844	Pcc11D	90	80	44
Pvc11	844	Pcc11E	58	106	39
Pvc11	844	Pcc11F	12	15	8
Pvc11	844	Pcc11G	0	28	7
Pvc11	844	Pcc11H	16	0	1
Pvc12	798	Pcc12A	366	361	181
Pvc12	798	Pcc12B	258	259	157
Pvc12	798	Pcc12C	225	246	151
Pvc12	798	Pcc12D	157	313	113
Pvc12	798	Pcc12E	0	14	5
Pvc13	1196	Pcc13A	470	441	234
Pvc13	1196	Pcc13B	346	342	203
Pvc13	1196	Pcc13C	251	232	151
Pvc13	1196	Pcc13D	188	217	115
Pvc13	1196	Pcc13E	141	173	101
Pvc13	1196	Pcc13F	24	0	9
Pvc14	602	Pcc14A	242	224	130
Pvc14	602	Pcc14B	175	187	104
Pvc14	602	Pcc14C	85	159	53
Pvc14	602	Pcc14D	0	25	6
Pvc15	560	Pcc15A	251	239	126
Pvc15	560	Pcc15B	164	194	99
Pvc15	560	Pcc15C	91	66	47
Pvc15	560	Pcc15D	53	96	38
Pvc15	560	Pcc15E	33	12	20
Pvc15	560	Pcc15F	16	23	14
Pvc15	560	Pcc15G	0	31	12
Pvc15	560	Pcc15H	11	0	4
Pvc15	560	Pcc15I	4	6	2
Pvc16	689	Pcc16A	283	267	180
Pvc16	689	Pcc16B	263	254	144
Pvc16	689	Pcc16C	42	9	11
Pvc16	689	Pcc16D	9	12	3
Pvc16	689	Pcc16E	0	10	3
Pvc16	689	Pcc16F	3	5	2
Pvc17	1282	Pcc17A	491	420	291
Pvc17	1282	Pcc17B	326	313	203
Pvc17	1282	Pcc17C	302	301	184
Pvc17	1282	Pcc17D	298	269	182
Pvc17	1282	Pcc17E	295	265	173
Pvc17	1282	Pcc17F	140	168	108
Pvc18	197	Pcc18A	859	569	59

Table 2. Statistics for the proto-gnathostome chromosomes.

Proto-gnathostome	Human genes	Chicken genes	Spotted gar genes	Elephant shark genes	Amphioxus orthologs
Pgc1	695	568	1127	676	496
Pgc2	335	251	288	306	252
Pgc3	280	42	227	229	194
Pgc4	541	471	564	611	473
Pgc5	413	349	391	436	335
Pgc6	227	26	0	0	61
Pgc7	774	702	258	802	569
Pgc8	395	328	362	356	278
Pgc9	741	600	640	651	627
Pgc10	268	216	279	298	165
Pgc11	318	245	281	329	207
Pgc12	142	85	93	16	60
Pgc13	498	421	528	515	333
Pgc14	132	0	201	49	117
Pgc15	792	635	1166	836	720
Pgc16	437	327	361	409	343
Pgc17	802	702	763	963	735
Pgc18	444	329	386	415	322
Pgc19	589	501	529	591	400
Pgc20	373	118	265	205	204
Pgc21	333	325	366	393	299
Pgc22	174	0	130	20	90
Pgc23	783	687	741	845	663
Pgc24	303	240	317	329	248
Pgc25	447	295	306	354	290
Pgc26	0	0	26	0	37
Pgc27	389	17	0	0	35
Pgc28	493	398	442	438	359
Pgc29	332	307	341	366	281
Pgc30	143	168	144	234	128
Pgc31	434	380	434	441	367
Pgc32	268	160	350	0	160
Pgc33	910	739	790	721	644
Pgc34	287	237	319	367	296
Pgc35	562	487	518	565	447
Pgc36	276	0	187	186	123
Pgc37	323	272	297	345	260
Pgc38	215	45	0	157	53
Pgc39	451	341	376	445	290
Pgc40	429	305	333	334	261
Pgc41	134	85	44	0	81
Pgc42	81	7	38	0	38
Pgc43	614	496	520	547	482
Pgc44	519	517	569	693	504
Pgc45	182	0	150	35	137
Pgc46	371	0	211	59	153
Pgc47	367	353	0	286	147
Pgc48	236	173	222	201	12
Pgc49	100	4	120	55	2

Table 3. Numbers of paralogs between proto-cyclostome chromosomes.

	Pcc1A	Pcc1B	Pcc1C	Pcc1D	Pcc1E	Pcc1F	Pcc2A	Pcc2B	Pcc2C	Pcc2D	Pcc2E	Pcc3A	Pcc3B	Pcc3C	Pcc3D	Pcc3E	Pcc3F	Pcc4A	Pcc4B	Pcc4C	Pcc4D	Pcc4E	Pcc4F	Pcc5A	Pcc5B
Pcc1A	39,19																								
Pcc1B	98,79	77,24																							
Pcc1C	130,95	64,65	13,9																						
Pcc1D	56,48	57,45	54,38	24,6																					
Pcc1E	34,36	46,44	26,33	16,25	5,4																				
Pcc1F	31,30	29,17	24,15	14,16	13,12	4,1																			
Pcc2A		0,3	1,0	1,0	1,2	0,1	20,29																		
Pcc2B		0,1	0,2	0,1	0,1		15,22	16,14																	
Pcc2C	0,2	2,1	1,1	0,1	1,0		13,20	20,11	10,11																
Pcc2D		0,2	0,1	0,1	2,1	1,1	20,14	10,5	12,6	3,1															
Pcc2E		2,11	3,3	2,1	0,1	0,2	8,10	3,9	6,3	3,2	22,18														
Pcc3A	2,4		1,3	2,4	0,2	1,0	2,1	1,3	1,1	0,1	0,2	55,16													
Pcc3B	0,1	1,0	0,1	1,2			0,1	2,4	1,2	2,2	0,6	45,43	25,12												
Pcc3C	2,4	1,1	1,5	2,3	1,2	0,1	1,2	0,1	0,1	1,0		67,50	45,39	11,12											
Pcc3D	5,4	1,0	1,2	1,0		0,1		2,1		0,2	0,1	45,45	37,24	38,32	14,5										
Pcc3E	0,1			0,1	0,1		2,2	1,3	1,2	1,1		22,17	9,14	15,26	16,13	16,3									
Pcc3F	2,2		0,1	1,1			0,1	0,2	0,1		0,1	8,13	12,18	15,15	6,9	0,2	1,3								
Pcc4A	5,4	0,1	3,0	2,2		1,0	0,2	1,0						0,1				12,4							
Pcc4B	1,2	0,1	0,2		0,1	0,1	1,1		0,2		0,1	0,1		0,1	0,1			7,12	3,3						
Pcc4C	0,1	0,2	1,2		1,1	1,0	0,1		0,1	1,0	4,4	1,0	1,1	0,3	1,2			13,13	6,3	21,16					
Pcc4D																					2,0				
Pcc4E							0,2							0,1						0,4		0,7			
Pcc4F																									
Pcc5A	2,5	0,4	1,1	1,1			1,2	7,2		1,0		2,4	2,0	1,3	0,1	1,2		0,1						15,8	
Pcc5B		1,1				0,1		0,2			1,0	13,5	1,0			1,1		1,0						24,21	19,8
Pcc5C		1,2	1,0	1,0							4,3	1,0	0,2							2,2				1,9	15,6
Pcc5D					0,1			0,1								0,2									
Pcc5E																								1,2	
Pcc5F																									
Pcc5G																								1,0	
Pcc6A	0,2	0,8	0,1	1,5	1,2		4,1		2,0		2,0	0,2	0,2	0,2	0,1	0,1	2,0							1,0	1,1
Pcc6B	3,3	1,1	1,0	2,4			2,2		3,2		0,1	1,0	2,0					4,4		0,1				0,1	4,1
Pcc6C	0,1	2,0	0,5	3,3			1,1		1,1		1,1			2,0			2,0	2,0							1,0
Pcc6D	0,1		0,1				0,1					0,1		0,2				1,0	0,1	0,1					
Pcc6E												9,2												1,4	1,0
Pcc6F	0,1			0,1			0,1						0,1											0,2	
Pcc6G																									
Pcc7A	0,1	2,2	1,0	1,1			2,2	0,1	1,0	0,1	1,0	0,1		0,2	1,0		0,1	0,1	0,1			0,1		0,1	2,2
Pcc7B	4,1	1,2	1,0	2,2	1,0		0,2	0,1		1,1		2,4	3,0	0,1	0,1			2,5		0,1				0,2	0,1

Table 4. Numbers of paralogs between proto-gnathostome chromosomes.

Pgc1	9,9,41,24																																																
Pgc2	93,57,102,103	5,2,5,4																																															
Pgc3	71,10,72,69	40,4,35,38	8,0,4,8																																														
Pgc4	132,122,186,177	64,52,80,88	90,15,95,88	11,12,26,60																																													
Pgc5	16,10,110,39	1,1,1,2	0,0,0,2	0,0,1,8	8,9,13,14																																												
Pgc6	4,1,0,0	1,0,0,0		1,1,0,0	33,1,0,0	3,0,0,0																																											
Pgc7	5,3,3,23	0,9,8,11	3,1,4,10	5,29,10,29	83,79,24,96	28,0,0,0	13,15,3,35																																										
Pgc8	4,2,111,12	0,0,0,2	1,0,2,1	3,3,7,4	42,40,61,53	20,0,0,0	153,136,62,140	2,5,4,12																																									
Pgc9	5,4,53,14	0,0,0,5	1,0,1,3	2,1,4,11	2,2,2,9	7,6,0,0	105,101,31,123	56,60,39,59	12,13,14,23																																								
Pgc10	1,1,40,7	0,0,0,2	1,0,1,0	0,0,1,3	0,0,0,3	5,3,0,0	43,48,11,56	27,30,31,34	91,97,82,104	5,3,5,9																																							
Pgc11	0,1,3,8	0,0,0,2	3,3,1,1	0,0,2,4	0,0,2,6		4,3,0,2	4,3,2,3	9,10,7,13	1,1,2,5	10,5,5,11																																						
Pgc12	0,0,2,0				0,0,1,0		1,0,0,0	1,0,1,0	0,4,4,0	2,3,0,0	22,22,20,1	0,1,0,0																																					
Pgc13	16,5,3,5	6,2,0,2	5,0,1,1	4,2,3,3	1,1,4,6	2,3,0,0	1,1,1,5	1,2,4,6	5,2,5,14	3,1,2,3	40,45,66,74	16,18,15,0	20,23,60,64																																				
Pgc14	1,0,1,1	0,0,1,0		0,0,0,1		1,0,0,0	0,0,0,2	0,0,1,0	1,0,0,0	0,0,5,0	12,0,20,1	4,0,6,0	44,0,65,13	4,0,6,1																																		
Pgc15	0,0,9,8	0,0,2,3	1,0,8,0	12,5,11,4	1,1,3,9		5,5,3,6	1,2,5,5	31,26,59,45	14,13,43,20	3,1,5,11	2,1,1,1	74,75,104,97	16,0,31,7	23,22,68,48																																	
Pgc16	2,2,4,7	1,1,0,1	0,0,0,1	8,2,2,4	0,2,4,10	6,6,0,0	0,0,2,7	0,1,2,1	21,24,23,30	11,14,15,17	1,2,3,3	2,2,1,0	39,49,57,64	16,0,21,2	107,136,195,176	4,6,9,15																																
Pgc17	5,2,4,13	0,0,3,4	1,1,2,6	2,1,9,12	1,0,2,6	1,0,0,0	0,2,0,14	1,0,1,1	2,3,5,6	1,1,0,8	3,3,4,9		3,3,10,14	0,0,0,1	97,95,107,121	35,50,46,52	16,11,20,31																															
Pgc18	8,2,1,5	0,0,1,1	1,1,3,3	1,2,2,3	0,0,1,1		0,1,1,2	0,0,1,1	0,0,1,1	0,1,1,6	1,1,2,1		6,4,4,5	0,0,1,1	46,46,51,54	20,24,25,41	134,149,137,178	6,7,9,25																													
Pgc19	11,5,4,7	1,2,2,2	1,0,3,3	2,1,4,5	0,0,2,1		1,2,1,11	0,0,0,4	13,13,11,13	0,0,0,2	0,1,1,2	0,0,0,1	5,5,4,6	7,0,4,2	2,1,10,5	0,0,1,4	107,124,127,180	71,75,80,101	17,19,15,17																														
Pgc20	5,3,4,1	1,2,4,1	2,1,5,0	3,2,6,0	0,0,1,2		10,0,1,4	3,0,0,1	2,0,1,1	2,0,0,2	1,0,0,1		0,0,0,3		0,0,6,3	0,0,2,0	42,31,49,50	43,22,41,29	101,56,115,78	13,0,10,3																													
Pgc21	3,3,5,4	0,1,2,2	0,0,0,2	1,3,2,2	2,2,3,3	1,0,0,0	3,1,3,4	1,1,1,1	1,1,1,2	0,1,0,3	0,0,0,3		1,1,1,6	1,0,2,0	4,6,14,9	4,4,2,2	2,2,2,5	2,2,2,3	1,2,1,3	0,0,16,0	10,13,20,16																												
Pgc22	0,0,2,0	1,0,1,0		0,0,1,1	1,0,2,0	1,0,0,0	1,0,0,0		1,0,1,0	1,0,1,0					3,0,0,0		3,0,3,0	1,0,2,0	0,0,4,1	0,0,2,0	28,0,35,7	0,0,1,0																											
Pgc23	3,2,4,8	1,1,1,7	2,0,2,5	3,3,3,13	2,1,2,5		3,2,4,5	0,3,0,9	5,2,2,12	0,0,2,2	2,0,2,6		3,3,8,13	0,0,2,0	5,5,9,6	1,0,1,1	3,3,4,10	0,0,0,2	0,0,1,3	1,0,6,3	113,125,136,140	38,0,40,3	21,24,30,44																										
Pgc24	1,0,2,1	0,0,0,1	1,0,1,0	1,0,3,2	1,0,1,2		1,1,0,7	0,0,1,1	3,2,2,4	1,1,3,5	0,0,0,1	1,1,1,0	3,2,3,4	2,0,1,0	2,2,4,5	0,0,2,3	2,2,2,5	2,1,1,2	3,4,2,0	1,0,0,1	40,39,49,64	16,0,23,1	117,109,131,141	9,6,11,19																									
Pgc25	0,1,2,3	1,0,0,1		3,1,1,2	2,1,1,5	1,0,0,0	2,1,0,3	0,0,0,3	1,1,1,7	0,1,1,2	0,0,0,6		2,3,0,2		5,3,5,7	3,2,1,4	11,3,2,3	1,0,0,1	1,1,1,4	0,0,0,2	0,0,0,2	1,0,0,0	94,98,114,116	41,44,49,46	4,4,10,15																								
Pgc26			0,0,1,0	0,0,1,0											0,0,1,0								0,0,6,0	0,0,2,0	0,0,7,0																								
Pgc27	1,0,0,0			2,0,0,0	2,0,0,0		1,1,0,0				1,0,0,0		1,0,0,0			2,0,0,0	2,0,0,0			1,0,0,0			24,0,0,0	9,0,0,0	29,0,0,0		3,0,0,0																						
Pgc28	2,3,6,4	0,0,0,1	0,0,1,0	0,1,1,1	3,2,3,5		1,3,0,5	1,0,0,2	1,1,1,7	2,0,3,0	0,0,2,2	2,0,4,0	1,0,0,3		0,1,2,5	0,1,1,5	2,2,2,8	1,1,2,1	4,1,2,4	3,1,2,1	2,2,2,3	0,0,1,0	13,3,3,5	0,0,2,7	1,1,1,3		20,1,0,0	15,11,11,12																					
Pgc29	2,2,5,6	0,0,0,1	1,0,1,1	0,0,1,4	3,4,6,1		3,4,0,3	1,0,0,2	0,0,1,0	0,0,2,0	0,0,1,1	0,0,4,0	0,0,0,9	0,0,2,0	0,1,2,3	0,1,1,5	2,2,3,5	0,0,2,1	0,0,1,0		2,3,1,5	1,0,1,0	10,3,3,10	3,1,1,2	3,1,2,3	0,0,1,0	19,0,0,0	74,80,113,120	8,8,11,13																				
Pgc30	0,0,0,2	0,0,1,0	0,0,0,3	0,0,2,2	0,1,0,1		1,1,0,3	0,0,0,2	1,0,1,2	0,0,1,0	0,0,2,0		0,0,0,4	0,0,1,1	0,1,3,6	1,2,1,1	0,1,0,5		0,1,1,3	0,0,1,3	1,2,1,2	2,0,0,0	1,2,2,29	1,1,0,18	2,2,1,24		11,0,0,0	41,53,33,50	37,44,27,39	2,1,1,3																			
Pgc31	0,0,2,2	1,0,1,1	0,0,0,1	1,1,2,8	2,1,2,1	1,0,0,0	0,0,1,3	0,1,1,2	5,6,3,8	1,2,3,2	2,2,2,1		1,0,0,8	0,0,1,0	3,1,6,9	1,1,2,1	6,4,23,10	1,1,2,2	3,2,4,6	6,0,1,2	0,2,1,1		1,2,2,8	0,0,0,1	0,1,1,2			1,0,2,3	0,0,0,2	0,0,0,1	6,9,11,12																		
Pgc32	0,0,2,0		0,0,1,0	0,1,2,0	0,0,3,0	1,0,0,0	1,0,3,0	0,1,1,0	2,1,2,0	1,1,5,0	1,1,2,0	0,0,1,0	1,1,1,0	0,0,1,0	5,0,25,0	0,0,1,0	3,1,10,0	1,0,5,0	4,1,1,0	2,0,1,0	1,1,0,0	0,0,1,0	0,0,2,0		0,0,2,0			1,1,2,0	0,0,2,0	0,0,3,0	57,33,65,0	4,1,11,0																	
Pgc33	2,3,6,5	4,0,3,2	2,0,4,1	10,2,7,6	1,3,0,7		1,3,1,15	1,1,1,4	6,5,7,8	5,4,5,2	1,1,3,3		2,2,1,8	0,0,2,7	16,7,23,9	2,2,4,4	8,5,5,12	8,12,38,6	5,8,41,6	3,0,26,3	0,1,0,7	7,0,0,0	12,4,2,9	6,2,2,9	0,2,3,9			3,2,7,4	3,4,1,0	1,1,3,4	128,124,143,157	56,23,45,0	26,20,27,24																
Pgc34	0,0,1,4	0,0,0,3	0,0,1,5	0,1,1,5	0,0,1,5		2,1,2,3	0,0,0,1	2,0,2,3	0,0,2,4	2,1,2,3		4,3,1,8		4,1,1,9	0,1,1,2	1,1,15,11	0,1,2,0	0,2,3,1	0,0,1,1	0,0,0,5	1,0,0,0	0,0,2,3	0,0,0,2	0,1,1,5	0,0,1,0		1,1,2,0	0,0,0,4		34,50,60,83	25,10,31,0	78,77,110,117	7,3,3,12															
Pgc35	3,3,3,8	0,0,1,3	2,0,1,1	3,3,7,10	0,0,4,8		0,0,1,9	0,0,0,2	0,0,1,4	1,1,4,2	1,1,1,2	0,0,0,1	5,3,3,4	1,0,2,2	6,3,7,11	4,0,1,2	2,5,17,21	0,0,7,1	0,0,2,6	3,0,0,0	0,0,0,2		1,1,1,6	0,1,0,4	3,3,2,2		1,0,0,0	1,2,1,6	1,1,1,1	0,2,2,3	1,0,0,1	0,0,4,0	67,65,63,79	32,25,39,35	21,23,26,22														
Pgc36	0,0,1,1	0,0,3,3		0,0,0,1	2,0,3,1		0,0,1,2	0,0,0,1	1,0,0,4		1,0,0,1		0,0,0,2	0,0,2,0	0,0,2,2	0,0,1,0	2,0,6,7	0,0,0,3	1,0,0,0		0,0,0,2		1,0,1,2		1,0,3,2		1,0,0,0		2,0,0,0		5,0,0,0	2,0,0,0	27,0,26,17	15,0,23,15	69,0,75,64	4,0,6,11													
Pgc37	3,4,2,3	0,0,1,1	1,0,1,1	0,0,0,5	1,0,0,3	0,1,0,0	1,0,0,4	1,0,2,4	1,1,1,8	3,1,1,3	1,0,0,2		2,2,8,3	0,0,2,0	3,4,13,10	0,1,1,4	3,3,4,2	1,0,1,1	3,4,2,4	1,0,0,3	1,1,0,2		1,2,1,3	0,0,0,3			2,0,0,0	5,2,4,3	3,1,3,2	1,1,1,2	0,2,0,0		4,3,2,2	4,0,0,4	79,82,87,94	20,0,27,25	6,10,16,16												
Pgc38	0,0,0,4	0,0,0,4			1,1,0,0	2,0,0,0		0,0,0,2	0,0,0,2	0,0,0,3	0,0,0,3	1,1,0,13		0,0,0,1	0,1,0,3	0,0,0,3	0,1,0,1	0,0,0,4	1,1,0,9		0,0,0,1		1,0,0,1		0,0,0,2			0,0,0,1	0,0,0,3	0,0,0,1	0,0,0,1	1,0,0,0	0,0,0,4	0,0,0,5	12,0,0,7	5,0,0,13	10,2,0,9	1,1,0,79											
Pgc39	2,2,6,6	0,0,0,2	0,0,1,1	2,2,2,2	2,2,2,3	2,0,0,0	3,4,7,9	4,4,3,3	1,0,1,9	1,2,1,4	0,1,2,5	1,1,2,0	2,4,3,11	1,0,0,2	0,0,2,6	0,0,0,1	1,1,1,15		1,0,0,4	0,1,1,4	1,1,4,3		4,1,3,4	1,2,2,6	2,1,3,3		1,0,0,0	6,1,0,0	1,0,0,4	0,0,0,1	0,0,1,1	2,1,0,0	2,0,3,6	1,2,2,3	1,1,2,3	0,0,0,3	2,1,1,2	2,2,0,1	10,11,15,14										
Pgc40	3,1,6,5	0,0,1,0	1,0,1,1	3,1,1,2	0,1,2,0		2,1,7,7	2,1,2,1	1,0,1,6	1,2,2,10	5,1,1,6	1,1,1,0	9,0,1,4	1,0,1,0	2,3,4,5	1,0,1,2	1,1,2,6	1,1,1,2	1,2,1,3	0,0,1,0	0,2,1,2	0,0,3,1	1,0,1,5	0,3,2,2	1,0,1,6			6,2,0,3		0,0,0,1	1,1,2,2	0,0,1,0	4,2,3,3	2,0,0,0	2,1,1,3	0,0,1,0	0,0,1,3	1,0,0,1	75,80,90,75	6,7,6,12									
Pgc41	0,0,1,0	0,1,0,0		0,1,1,0	0,1,0,0		0,1,1,0		0,1,0,0						0,1,0,0	0,1,0,0	1,2,1,0	0,1,0,0	1,0,0,0		0,1,0,0		10,3,0,0	2,1,0,0			7,0,0,0			0,1,0,0	2,1,1,0		3,0,1,0	1,0,0,0				0,1,0,0	19,16,16,0	13,6,9,0	2,2,0,0								
Pgc42									1,0,0,0						0,0,1,0	0,0,1,0							1,0,0,0										3,0,0,0						20,0,1,0	17,0,2,0	3,0,1,0								
Pgc43	1,1,1,6	1,0,1,0		0,0,0,3	5,4,2,5		3,1,0,2	1,0,1,4	20,7,40,2	0,0,0,2	3,4,1,2		6,5,3,4	0,0,1,0	1,1,3,9	3,2,1,1	3,3,3,12	0,0,1,2	1,1,1,8	1,1,1,2	1,0,1,4		2,1,2,5	4,0,0,3	1,1,1,4		2,0,0,0	3,2,1,4	1,1,2,2	1,2,1,2	1,1,4,3	0,0,1,0	2,1,3,4	0,0,2,1	1,1,4,1	0,0,1,0	0,0,0,2	0,0,0,14	1,1,1,2	1,0,1,3	0,2,0,0		7,10,8,25						
Pgc44	2,2,31,5	3,0,0,1	1,0,0,2	1,0,1,2	3,1,4,7	1,1,0,0	1,4,6,5	2,1,17,4	3,3,9,2	1,1,36,10	2,2,1,3	0,0,1,0	1,3,4,5	0,0,1,1	1,2,16,20	2,1,5,9	3,3,5,6	1,0,1,4	3,0,2,2	1,0,0,1	1,2,2,3	1,0,2,0	2,2,4,20	3,0,3,3	1,1,2,5		1,0,0,0	2,3,1,5	1,3,0,7	1,1,0,2	0,0,3,5	0,0,2,0	5,1,1,6	1,0,2,6	2,3,0,3	0,0,2,1	1,1,1,0	0,0,0,2	0,0,10,18	1,2,2,1	0,1,0,0		129,156,124,156	6,16,12,19					
Pgc45			1,0,0,1	0,0,0,1	0,0,1,0				8,0,8,2		1,0,1,0		1,0,2,0		0,0,1,0	1,0,1,0	1,0,1,3						1,0,1,0	4,0,0,0	1,0,1,0			1,0,2,1	0,0,2,0	0,0,1,0					0,0,0,1				5,0,0,2	1,0,0,2		2,0,0,0	43,0,39,9	44,0,31,12	1,0,1,0				
Pgc46	1,0,3,0	1,0,1,0	1,0,0,0	1,0,0,0	1,0,1,2		0,0,0,1		9,0,15,1	0,0,0,1	1,0,0,1	1,0,0,0	1,0,6,0	0,0,1,0	2,0,1,1	0,0,1,0	2,0,1,3	1,0,1,1	2,0,2,0	2,0,1,0	0,0,1,0		1,0,1,0	1,0,0,0			1,0,0,0	0,0,1,1	2,0,3,1	1,0,0,0	0,0,1,0	1,0,2,0	2,0,3,0	1,0,1,1	2,0,1,0	1,0,0,0		0,0,0,1	1,0,1,2	1,0,3,0			59,0,73,23	43,0,49,23	15,0,18,3	3,0,5,1			
Pgc47	2,1,0,2	0,0,0,1	2,0,0,2	1,3,0,0	0,1,0,5		1,2,0,5	1,1,0,0	40,34,0,27	14,11,0,12	2,2,0,4		3,4,0,31	2,0,0,2	6,9,0,9	9,22,0,15	3,5,0,9	2,1,0,1	1,3,0,5	2,0,0,3	0,3,0,7		2,2,0,4	4,2,0,1	2,2,0,0		2,0,0,0	0,0,0,3	1,1,0,3	1,3,0,2	1,2,0,2	1,2,0,0	4,5,0,4	1,1,0,1	4,3,0,1	1,0,0,0	1,2,0,3	0,0,0,4	1,2,0,7	1,2,0,4	0,1,0,0	1,0,0,0	1,1,0,1	1,3,0,19		15,0,0,0	7,16,0,19		
Pgc48	1,1,1,3	0,0,2,1	1,0,0,1	1,1,4,5	0,0,0,1		0,0,0,2		1,1,0,6	0,0,1,2	1,0,0,0		1,0,4,2	0,0,0,1	2,1,56,18	3,1,2,4	0,3,4,6	0,0,1,1	1,1,3,2	1,0,2,1	2,3,2,1	0,0,1,0	5,4,4,6	1,1,0,0	3,2,3,0	0,0,1,0	1,0,0,0	0,2,1,4	3,2,2,0	1,3,2,0	0,0,1,2	2,2,27,0	1,1,1,10	0,0,0,2	5,2,3,3	0,0,0,1	0,1,0,1	0,0,0,5	1,1,3,1	1,1,3,2	0,1,0,0		0,0,1,2	1,1,4,1	1,0,3,0	18,0,0,0	39,48,0,45	6,6,11,11	
Pgc49	0,0,3,6		1,0,0,0		0,0,2,5			0,0,2,0	0,0,0,5				0,0,0,1		0,0,4,0			0,0,0,1	0,0,0,1				1,0,0,0		1,0,0,1			0,0,0,1	1,0,2,1			0,0,3,0		0,0,0,1	3,0,0,2			0,0,0,22	0,0,1,12							4,0,0,1	14,1,0,0	14,0,3,0	1,0,0,1
	Pgc1	Pgc2	Pgc3	Pgc4	Pgc5	Pgc6	Pgc7	Pgc8	Pgc9	Pgc10	Pgc11	Pgc12	Pgc13	Pgc14	Pgc15	Pgc16	Pgc17	Pgc18	Pgc19	Pgc20	Pgc21	Pgc22	Pgc23	Pgc24	Pgc25	Pgc26	Pgc27	Pgc28	Pgc29	Pgc30	Pgc31	Pgc32	Pgc33	Pgc34	Pgc35	Pgc36	Pgc37	Pgc38	Pgc39	Pgc40	Pgc41	Pgc42	Pgc43	Pgc44	Pgc45	Pgc46	Pgc47	Pgc48	Pgc49
