# Supplementary material for: Reconstruction of proto-vertebrate, proto-cyclostome and proto-gnathostome genomes provides new insights into early vertebrate evolution
Source: Nat Commun. 2021 Jul 23;12:4489. doi: 10.1038/s41467-021-24573-z (PMC8302630; doi:10.1038/s41467-021-24573-z)
Supplement: Supplementary file 6 — Reporting Summary [file 41467_2021_24573_MOESM6_ESM.pdf]

nature research

Corresponding author(s): Byrappa Venkatesh, Aoife McLysaght

Last updated by author(s): Jun 22, 2021

Reporting Summary

Nature Research wishes to improve the reproducibility of the work that we publish. This form provides structure for consistency and transparency in reporting. For further information on Nature Research policies, see Authors & Referees and the Editorial Policy Checklist .

Statistics

For all statistical analyses, confirm that the following items are present in the figure legend, table legend, main text, or Methods section.

n/a	Confirmed	
☒	☐	The exact sample size (*n*) for each experimental group/condition, given as a discrete number and unit of measurement
☒	☐	A statement on whether measurements were taken from distinct samples or whether the same sample was measured repeatedly
☒	☐	The statistical test(s) used AND whether they are one- or two-sided<br>*Only common tests should be described solely by name; describe more complex techniques in the Methods section.*
☒	☐	A description of all covariates tested
☒	☐	A description of any assumptions or corrections, such as tests of normality and adjustment for multiple comparisons
☒	☐	A full description of the statistical parameters including central tendency (e.g. means) or other basic estimates (e.g. regression coefficient) AND variation (e.g. standard deviation) or associated estimates of uncertainty (e.g. confidence intervals)
☒	☐	For null hypothesis testing, the test statistic (e.g. *F*, *t*, *r*) with confidence intervals, effect sizes, degrees of freedom and *P* value noted<br>*Give P values as exact values whenever suitable.*
☒	☐	For Bayesian analysis, information on the choice of priors and Markov chain Monte Carlo settings
☒	☐	For hierarchical and complex designs, identification of the appropriate level for tests and full reporting of outcomes
☒	☐	Estimates of effect sizes (e.g. Cohen's *d*, Pearson's *r*), indicating how they were calculated

Our web collection on statistics for biologists contains articles on many of the points above.

Software and code

Policy information about availability of computer code

Data collection	No software was used for data collection.
Data analysis	FALCON Assembler v0.3.0: to generate contig-level genome assembly<br>Redundancy removal pipeline: to remove redundant contigs<br>Pilon v1.20: genome assembly error correction using Illumina reads<br>HiRise: for scaffolding contigs using Chicago and Hi-C libraries<br>PBJelly from PBSuite v15.8.24: gap-filling in scaffolds using error-corrected PacBio reads<br>RepeatModeler v1.0.10: prediction of de novo repeat library<br>TEclass v2.1.3: to classify predicted repeats<br>RepeatMasker v4.0: to mask repeats in the genome using the repeat library<br>MAKER v2.31.8: genome annotation<br>AUGUSTUS v3.2.1: ab initio gene prediction<br>BLAST+ v2.2.8 and v2.5.0: for homology-based searches<br>cdhit v4.6.1: program for clustering nucleotide and protein sequences.<br>BUSCO v2.0: to evaluate completeness of genome assembly<br>BLAT v35: to align RNA-seq data to genome<br>Ancestral genome reconstruction software<br>HMMER v3.1b2 and v3.3.2 for multiple alignment<br>RAxML v8.2.11 and v8.2.12 for gene tree analysis

For manuscripts utilizing custom algorithms or software that are central to the research but not yet described in published literature, software must be made available to editors/reviewers. We strongly encourage code deposition in a community repository (e.g. GitHub). See the Nature Research guidelines for submitting code & software for further information.

Data

Policy information about availability of data

All manuscripts must include a data availability statement. This statement should provide the following information, where applicable:

- Accession codes, unique identifiers, or web links for publicly available datasets
- A list of figures that have associated raw data
- A description of any restrictions on data availability

The Japanese lamprey and elephant shark genome sequences generated in this study have been submitted to GenBank under the BioProject accession numbers PRJNA575627 and PRJNA575613, respectively. The reconstruction dataset is available as Supplementary Data 1.

Field-specific reporting

Please select the one below that is the best fit for your research. If you are not sure, read the appropriate sections before making your selection.

☐ Life sciences ☐ Behavioural & social sciences ☒ Ecological, evolutionary & environmental sciences

For a reference copy of the document with all sections, see nature.com/documents/nr-reporting-summary-flat.pdf

Ecological, evolutionary & environmental sciences study design

All studies must disclose on these points even when the disclosure is negative.

Study description	We sequenced the elephant shark and Japanese lamprey genomes and used these for reconstruction of the ancestral vertebrate genome as well as to identify ohnologs in human which may have implications for understanding human genetic diseases.
Research sample	Elephant shark (Callorhinchus milii): Chondrichthyes (cartilaginous fishes). Genomic DNA from the testis of a single individual caught in Tasmania, Australia. This sample was also used for our previous publication: Venkatesh et al. Elephant shark genome provides unique insights into gnathostome evolution. Nature (2014).<br>Japanese lamprey (Lethenteron japonicum): Cyclostomata (jawless vertebrates). Genomic DNA from fully mature testis of a single adult individual caught in Hokkaido, Japan. This sample was also used for our previous publication: Mehta et al. Evidence for at least six Hox clusters in the Japanese lamprey (Lethenteron japonicum). Proc. Natl. Acad. Sci. USA. (2013)
Sampling strategy	Testis of a single individual was used for genomic DNA extraction for both elephant shark and Japanese lamprey.
Data collection	N.A.
Timing and spatial scale	Elephant shark tissues collected from Tasmania, Australia in 2004.<br>Japanese lamprey tissues collected from Hokkaido, Japan in 2010.
Data exclusions	No data were excluded.
Reproducibility	N.A.
Randomization	N.A.
Blinding	N.A.

Did the study involve field work? ☐ Yes ☒ No

Reporting for specific materials, systems and methods

We require information from authors about some types of materials, experimental systems and methods used in many studies. Here, indicate whether each material, system or method listed is relevant to your study. If you are not sure if a list item applies to your research, read the appropriate section before selecting a response.

Materials & experimental systems

n/a	Involved in the study	
☒	☐	Antibodies
☒	☐	Eukaryotic cell lines
☒	☐	Palaeontology
☐	☒	Animals and other organisms
☒	☐	Human research participants
☒	☐	Clinical data

Methods

n/a	Involved in the study	
☒	☐	ChIP-seq
☒	☐	Flow cytometry
☒	☐	MRI-based neuroimaging

Animals and other organisms

Policy information about studies involving animals; ARRIVE guidelines recommended for reporting animal research

Laboratory animals	*For laboratory animals, report species, strain, sex and age OR state that the study did not involve laboratory animals.*
Wild animals	An elephant shark male individual was captured from Tasmania, Australia.<br>Japanese lamprey was captured from Hokkaido, Japan.
Field-collected samples	*For laboratory work with field-collected samples, describe all relevant parameters such as housing, maintenance, temperature, photoperiod and end-of-experiment protocol OR state that the study did not involve samples collected from the field.*
Ethics oversight	No ethical approval was required as elephant shark is commercially exploited in Australia for human consumption. Likewise, Japanese lamprey is also commercially exploited in rivers near Sapporo, Hokkaido, Japan.

Note that full information on the approval of the study protocol must also be provided in the manuscript.
